# Discovery and biosynthesis of macrophasetins from the plant pathogen fungus *Macrophomina phaseolina*

**DOI:** 10.3389/fmicb.2022.1056392

**Published:** 2022-11-14

**Authors:** Cui Yu, Lin Chen, Yang Le Gao, Jia Liu, Pei Lin Li, Ming Liang Zhang, Qin Li, Huai Dong Zhang, Man Cheng Tang, Li Li

**Affiliations:** ^1^Engineering Research Center of Industrial Microbiology (Ministry of Education) and College of Life Sciences, Fujian Normal University, Fuzhou, China; ^2^State Key Laboratory of Microbial Metabolism, Joint International Research Laboratory of Metabolic and Developmental Sciences, School of Life Sciences and Biotechnology, Shanghai Jiao Tong University, Shanghai, China; ^3^Zhangjiang Institute for Advanced Study, Shanghai Jiao Tong University, Shanghai, China

**Keywords:** 3-Decalinoyltetramic acid, bioinformatics analysis, *Macrophomina phaseolina*, heterologous expression, genome mining

## Abstract

3-Decalinoyltetramic acids (DTAs) are a class of natural products with chemical diversity and potent bioactivities. In fungal species there is a general biosynthetic route to synthesize this type of compounds, which usually features a polyketide synthase-nonribosomal peptide synthetase (PKS-NRPS) and a lipocalin-like Diels-Alderase (LLDAse). Using a synthetic biology approach, combining the bioinformatics analysis prediction and heterologous expression, we mined a PKS-NRPS and LLDAse encoding gene cluster from the plant pathogenic fungus *Macrophomina phaseolina* and characterized the cluster to be responsible for the biosynthesis of novel DTAs, macrophasetins. In addition, we investigated the biosynthesis of these compounds and validated the accuracy of the phylogeny-guided bioinformatics analysis prediction. Our results provided a proof of concept example to this approach, which may facilitate the discovery of novel DTAs from the fungal kingdom.

## Introduction

**Figure 1:**
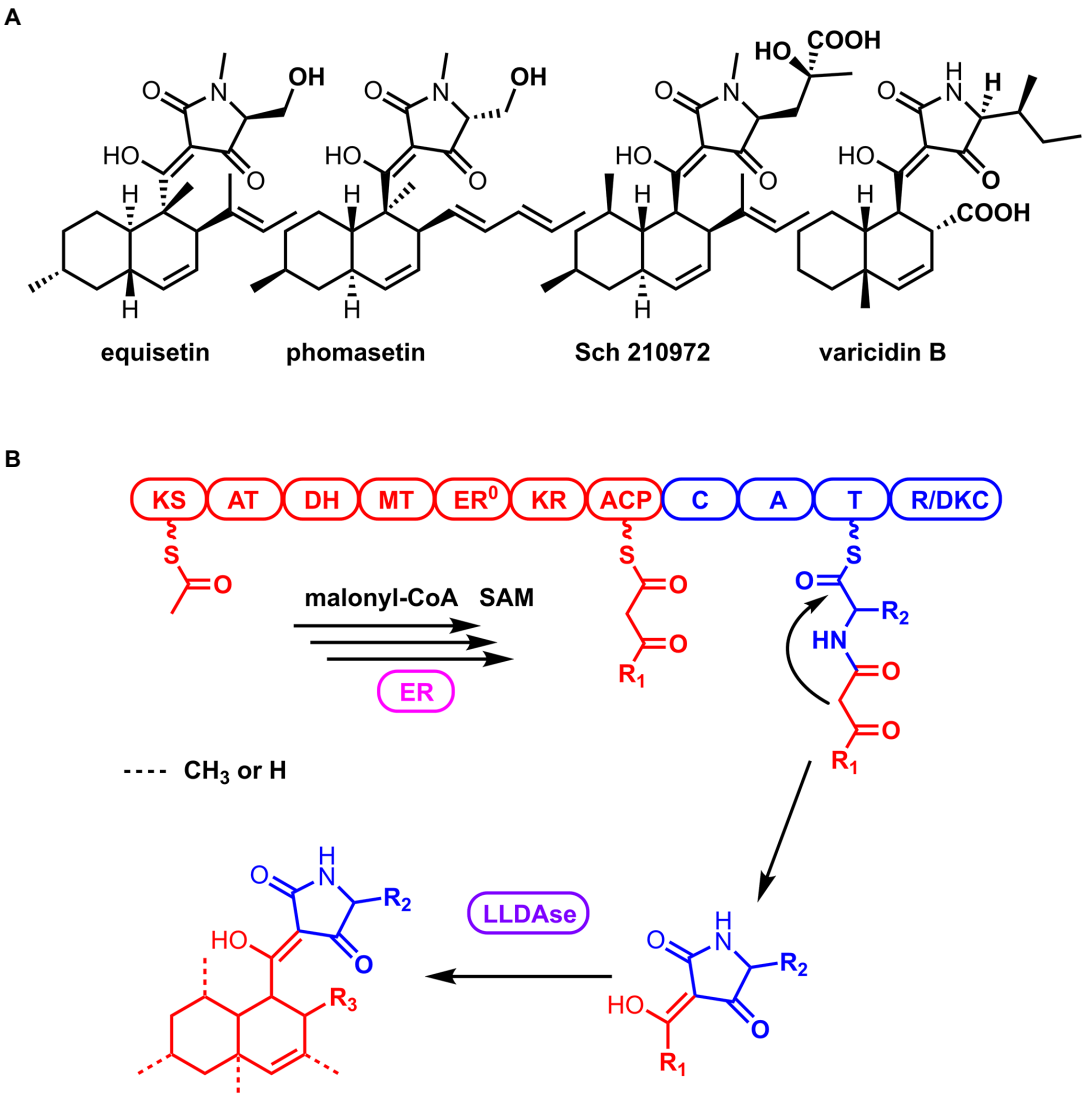
Structures of the representative 3-Decalinoyltetramic acids (DTAs; **A**) and the general biosynthetic route of DTAs in fungi **(B)**.

3-Decalinoyltetramic acids (DTAs) are a class of natural products isolated from various organisms, which feature a tetramate (pyrrolidine-2,4-dione) unit connected to a decalin fragment with multiple chiral centers (Figure 1A; Schobert and Schlenk, 2008; Jiang et al., 2020). Due to their structural complexity and potent bioactivity, this group of compounds has been attracting a great deal of attention for their chemical synthesis and biosynthesis (Yin et al., 2013; Kauhl et al., 2016; Li et al., 2017; Kato et al., 2018; Kauhl et al., 2018; Fan et al., 2019; Tan et al., 2019; Chi et al., 2021). Recent biosynthetic studies have revealed that there is a general route to biosynthesize this type of compounds in fungi (Mo and Gulder, 2021). The carbon backbone is derived from a bimodular megasynthase, known as polyketide synthase (PKS)-nonribosomal peptide synthetase (NRPS), assembly line (Figure 1B). The PKS module is typically a highly reducing PKS that iteratively synthesize the polyketide portion. And its dissociated enoylreductase (ER) partner catalyzes the selective enoylreduction during different cycles to furnish the diene and dienophile that is ready for the decalin construction. The NRPS module selectively activates a specific amino acid (AA) and catalyzes the condensation of this activated amino acid and the mature polyketide to form an amide bond. Then the product of the assembly line is released by the C-terminal reductase/Dieckmann cyclase (R/DKC) domain, through Dieckmann cyclization to generate the tetramic acid. The decalin moiety is constructed off the assembly line through intramolecular Diels-Alder (IMDA) reaction that catalyzed by a lipocalin-like Diels-Alderase (LLDAse) in a regioselective and stereoselective manner. Other tailoring modifications, such as methylation and hydroxylation, further diverse the chemical structures. Recent genome sequencing efforts have identified that fungi encodes a significant number of biosynthetic gene clusters (BGCs) containing PKS-NRPS and LLDAse encoding genes. However, many of them have not been exploited or characterized to target DTAs.

*Macrophomina phaseolina* is known as a plant pathogenic fungus, which is capable of causing disease in more than 500 different plants including economically important crops (Kaur et al., 2012; Ijaz et al., 2013; Degani et al., 2020). This fungal species mainly causes severe charcoal rot, which is thought to be the second crucial soybean disease in the United States (Luna et al., 2017). Recent studies revealed that this fungal species is also a potential rich resource for structurally diverse bioactive metabolites, evidenced by the fact that there are more than 40 genes encoding PKSs, NRPSs, and their hybrids in its genome (Islam et al., 2012). Novel compounds have been reported from *M. phaseolina* in the past few years, exemplified by the dipeptide serine-glycine-betaine (Singh et al., 2021), the macrolide phaseolide A (Morishita et al., 2020), and the phytotoxic cyclopentenones phaseocyclopentenones (Masi et al., 2021). Recently, we also reported the discovery of novel polyketide-amino acid hybrid compounds from *M. phaseolina* through genome mining (Gao et al., 2021). However, to date, no compounds belonging to the DTA family has been reported from this species.

To facilitate the discovery of novel bioactive secondary metabolites from fungal species, a set of new methods have been developed in recent years, including heterologous expression and transcription factor activation (Bauman et al., 2021). Most of these methods are focusing on the rapid discovery of novel natural products from diverse fungal species without accurate structural prediction, which means some of the natural products produced are known ones. Recently, Oikawa group reported the phylogeny-based bioinformatics analysis of fungal PKS-NRPS containing BGCs and linked a given BGC to a particular family of natural products (Minami et al., 2020). Inspired by their work, we reasoned that a synthetic biology approach combining bioinformatics prediction and heterologous expression is a promising way to mine novel DTAs from fungal species with improved accuracy. Here, we reported the discovery and biosynthesis of novel DTAs, macrophasetins, from *M. phaseolina* as a proof of concept to this approach.

## Materials and methods

### Strains, plasmids, and cultivation conditions

*Macrophomina phaseolina* MS6 was cultivated in PDA (potato dextrose agar, BD) or PDB (potato dextrose broth, BD) at 28°C (Gao et al., 2021). *Escherichia coli* DH5α were grown in Luria-Bertani media at 37°C for the standard DNA manipulation. *Saccharomyces cerevisiae* BJ5464-NpgA (*MATα ura3-52 his3-Δ200 leu2-Δ1 trp1 pep4::HIS3 prb1 Δ1.6R can1 GAL*) was used for expression plasmids construction (Wang et al., 2020). *Aspergillus nidulan*s A1145 was cultured at 30°C in CD media (1% glucose, 5% 20 × nitrate salts, 0.1% trace elements, and 2% agar for solid media) and used as the host of heterologous expression (Li et al., 2018). Plasmids pYTU, pYTP, and pYTR were used as heterologous expression vectors (Li et al., 2016).

### Bioinformatics analysis

Phylogenetic analysis was conducted using maximum likelihood method with MEGA 6 (Tamura et al., 2013). The Phyre2 web portal was used for protein modeling, prediction and analysis (Kelly et al., 2015). The three-dimensional structures of MpsD based on the template CghA (PDB ID: 6KAW) was selected. The protein was 3-D protonated and energy minimized using default parameters of MOE 2014 (Molecular Operating Environment, version 2014.0901). The MMFF94 (Wahl et al., 2019) force field was applied to minimize the initial structures to yield the lowest energy 3D conformation. The binding site was determined based on the PLB (Propensity for Ligand Binding) score in the Site Finder module. Besides, the structure of **2** was constructed by ChemDraw software and then optimized with MOE. MOE-docking was employed to investigate the binding modes of MpsD and compound **2**. The best poses were kept for binding mode analysis.

### Heterologous expression of *mps* in *Aspergillus nidulans*

Vectors of pYTU, pYTP, and pYTR were digested with *Pac*I and *Swa*I (New England Biolabs) for the expression plasmids construction. The genes in the *mps* cluster were amplified by PCR with Q5 High-Fidelity DNA polymerases (New England Biolabs) and cloned into vectors by recombination in yeast. The *mps* genes were amplified using PCR with the genomic DNA of *M. phaseolina* as the template. The *mpsA* was obtained in three pieces using primers UmpsAF1/R1, UmpsAF2/R2 and UmpsAF3/R3; the amyB promoter was amplified from pYTP with primers of AmyB-pYTU-F/R; *mpsG* was obtained by PCR with primers of UmpsGF/R. The five DNA fragments and pYTU digested with *Pac*I and *Swa*I were co-transformed into *S. cerevisiae* BJ5464-NpgA for assembly, leading to plasmid pYL081. The *mpsD* and *mpsF* were also amplified using the primers of PmpsDF/R and RmpsFF/R, respectively, and cloned into pYTP and pYTR by yeast homologous recombination, leading to pYL082 and pYL083. Yeast transformation was performed using Frozen-EZ Yeast Transformation II Kit (Zymo Research). The primers used for the heterologous expression are listed in Supplementary Table S1. The maps of pYL081, 082 and 083 were shown in Supplementary Figure S1.

The DNA transformation of *A. nidulans* A1145 was carried out as described by Li et al. (2016). The liquid CD-ST media (1 L: 20 g Starch, 20 g Peptone, 50 ml 20× Nitrate salts, 1 ml Trace elements, pH 6.5) was used for the production of secondary metabolites in heterologous expression.

### Characterization of compounds produced by heterologous expression

*A. nidulans* harboring *mps* genes were grown in CD-ST for 5 days and then extracted with ethyl acetate twice. The organic phase was dried by speed Vacuum and dissolved in methanol for analysis. LC–MS analyses were performed on Thermo Scientific U3000/LCQ Fleet with Phenomenex Luna C18 column (3 μm, 2.0 × 150 mm). LC analyses were achieved with a linear gradient of 5–95% CH_3_CN-H_2_O in 30 min followed by 95% CH_3_CN for 5 min with a flow rate of 0.25 ml/min. For isolation of compounds, ethyl acetate extract from 10 L culture was evaporated by Buchi Rotavapor and the crude extracts were injected to Combi-Flash system (Teledyne Isco) with a reversed-phase C18 column for initial separation. Fractions containing the target compounds were used for further purification by HPLC with a C18 column of Phenomenex Luna (5 μm, 10 × 250 mm). 1D and 2D NMR spectra were obtained on Bruker AVANCE III HD 600 MHz NMR spectrometer to elucidate the chemical structures of compounds.

### X-ray single-crystal diffraction for 3

X-ray single-crystal diffraction was performed on an Oxford Gemini S Ultra single-crystal diffractometer. A suitable crystal was selected and subjected to λ(MoKα) = 0.71073 Å at 273.15 K. The structure was determined using the direct method with Olex2 and refined with full-matrix least-squares calculations on F2 using Olex2.

## Results and discussion

### Bioinformatics analysis and product prediction of the *mps* cluster

**Figure 2:**
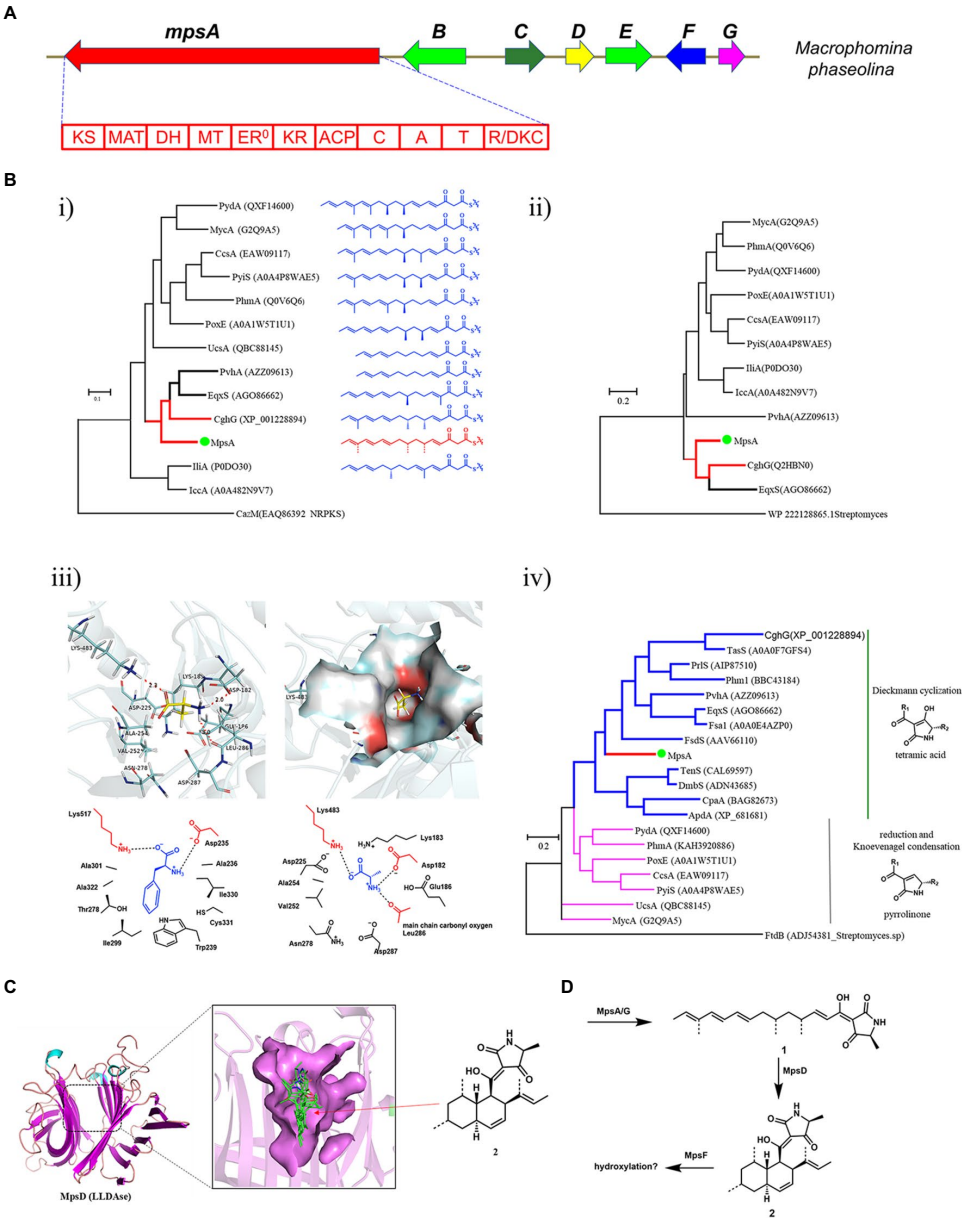
Bioinformatics analysis of the *mps* cluster. **(A)** The *mps* cluster identified from *M. phaseolina*. *mpsA* encodes PKS-NRPS, *mpsB* encodes transcription factor, *mpsC* encodes transporter, *mpsD* encodes LLDAse, *mpsE* encodes transcription factor, *mpsF* encodes cytochrome P450, *mpsG* encodes trans-acting ER. **(B)** Bioinformatics analysis of the domains identified from MpsA. (i) The phylogenetic tree of KS domains; (ii) The phylogenetic tree of MT domains; (iii) Structure-based prediction of the substrate of MpsA A domain; (iv) The phylogenetic tree of R/DKC domains. **(C)** Structure-based prediction of the product of MpsD. **(D)** The predicted metabolites encoded by *mps* cluster.

Using the PKS-NRPS Fsa1 and LLDAse Fsa2 as the search query (Kato et al., 2015), we identified a compact BGC, *mps* cluster, from the genome of *M. phaseolina* (Figure 2A). The *mps* cluster encodes a PKS-NRPS (*mpsA*, MPH_07623), a putative LLDAse (*mpsD*, MPH_07627), a cytochrome P450 (*mpsF*, MPH_07629), a trans-acting ER (*mpsG*, MPH_07630), and two putative transcription factors (*mpsB*, MPH_07625 and *mpsE*, MPH_07628) together with a putative transporter (*mpsC*, MPH_07626). To explore the unknown natural product encoded by the *mps* gene cluster, we first did the bioinformatics analysis of the biosynthetic genes to predict the potential structure to assess whether it’s a novel DTA.

We first analyzed the PKS-NRPS MpsA to predict the linear backbone of the final product. This enzyme contains 10 domains, which are, from the *N*-terminal to the *C*-terminal, ketosynthase (KS), acyltransferase, dehydratase, methyltransferase (MT), ketoreductase, acyl carrier protein, condensation, adenylation (A), peptide carrier protein, and R/DKC domains. For the polyketide portion, since the KS domain iteratively functions in each polyketide chain elongation step, we hypothesized that the closer the phylogenetic relationship of the KS domain the closer the structure of the polyketide chain produced by the PKS portion. Therefore, we constructed a phylogenetic tree of the KS domains identified from the reported fungal PKS-NRPS proteins by using the KS domain of CazM (Winter et al., 2015), a non-reducing PKS, as a root. As shown in Figure 2B, the structures of the polyketide products produced by different fungal PKS-NRPSs indeed showed correlation to the phylogenetic relationship of the KS domains. As the KS domain of MpsA is phylogenetically close to the KS domain of CghG (Sato et al., 2015), a PKS-NRPS from the Sch 210972 biosynthetic pathway, we proposed that the structure of the polyketide chain produced by MpsA is an octaketide with four olefinic bonds, similar to the polyketide chain produced by CghG. Furthermore, the MT domain of MpsA is also phylogenetically close to the MT domain of CghG (Figure 2B). Therefore, we proposed that the polyketide chain produced by MpsA would contain the similar methylation pattern as in Sch 210972.

Next, we analyzed the A domain of the NRPS portion, which is responsible for the AA activation and loading. Because the substrate specificity of A domains is dedicated by the AA residues surrounding the active site pocket (known as 10 AA code; Stachelhaus et al., 1999; Challis et al., 2000), we then did the sequence alignment of MpsA A domain with other fungal PKS-NRPS A domains, and identified the 10 AA code of MpsA A domain (Supplementary Table S6). However, compared to the known 10 AA code of other fungal NRPS A domains, the specific AA substrate of MpsA A domain could not be concluded. Therefore, to predict the AA substrate, a homology model of MpsA A domain was constructed using the structure of PheA (Conti et al., 1997; PDB ID: 1AMU) as a guide. When docking different AAs into the active site pocket we found that l-alanine is the probable substrate of MpsA A domain (Figure 2B).

Then, the bioinformatics analysis of the terminal R/DKC domain of MpsA was carried out, which is responsible for the product release from PKS-NRPS assembly line. Phylogenetic analysis revealed that the R/DKC domains from fungal PKS-NRPSs were separated into two groups (Figure 2B). The R/DKC domains catalyzing the Dieckmann cyclization to release the product as a tetramic acid fall into one group. And the R/DKC domains catalyzing the reduction release then followed by Knoevenagel condensation to yield a pyrrolinone product falling into the other group. The MpsA R domain phylogenetically falls into the group of R/DKC domains catalyzing the formation of a tetramic acid. Therefore, combined the analysis results of different domains of MpsA, we proposed that the linear product of MpsA could be the tetramic acid **1** (Figure 2D).

Last, we analyzed the enzymes involved in the post modification steps off the PKS-NRPS assembly line. Phylogenetic analysis of the reported LLDAses identified from different fungal species showed that they also clade into two groups. Group 1 LLDAses catalyze the IMDA reaction on tetramic acid substrates. Group 2 LLDAses catalyze the IMDA reaction on pyrrolinone substrates. The putative LLDAse MpsD clades in the group 1 LLDAses, suggesting it acts on a tetramic acid substrate which is consistent with the product prediction of MpsA. To further predict the product of MpsD, a homology model of MpsD was constructed using the structure of CghA (Sato et al., 2021; PDB ID: 6KAW) as a guide (Figure 2C). Docking studies with different IMDA reaction products of **1** were carried out. The results showed that the best matching product is **2** (structure shown in Figure 2D), which is the endo IMDA reaction product of **1** and contains a *trans* decalin ring. However, due to the diverse functions of P450s and limited structural information of fungal P450s, the function of P450 MpsF could not be precisely predicted. Since MpsF is phylogenetically close to PoxM (Sato et al., 2017; Supplementary Figure S21), a P450 from the oxaleimide biosynthetic pathway, we proposed that MpsF might catalyze the hydroxylation on **2**.

Based on the bioinformatics analysis results of the biosynthetic enzymes, we predicted the unknown natural product encoded by the *mps* cluster is a hydroxylated derivative of DTA **2** (Figure 2D). After searching in the compound databases, such as SciFinder, we did not find a known natural product with the same structural scaffold of **2**, suggesting the metabolite encoded by *mps* cluster could be a novel DTA.

### Heterologous expression of the *mps* cluster and characterization of the products

**Figure 3:**
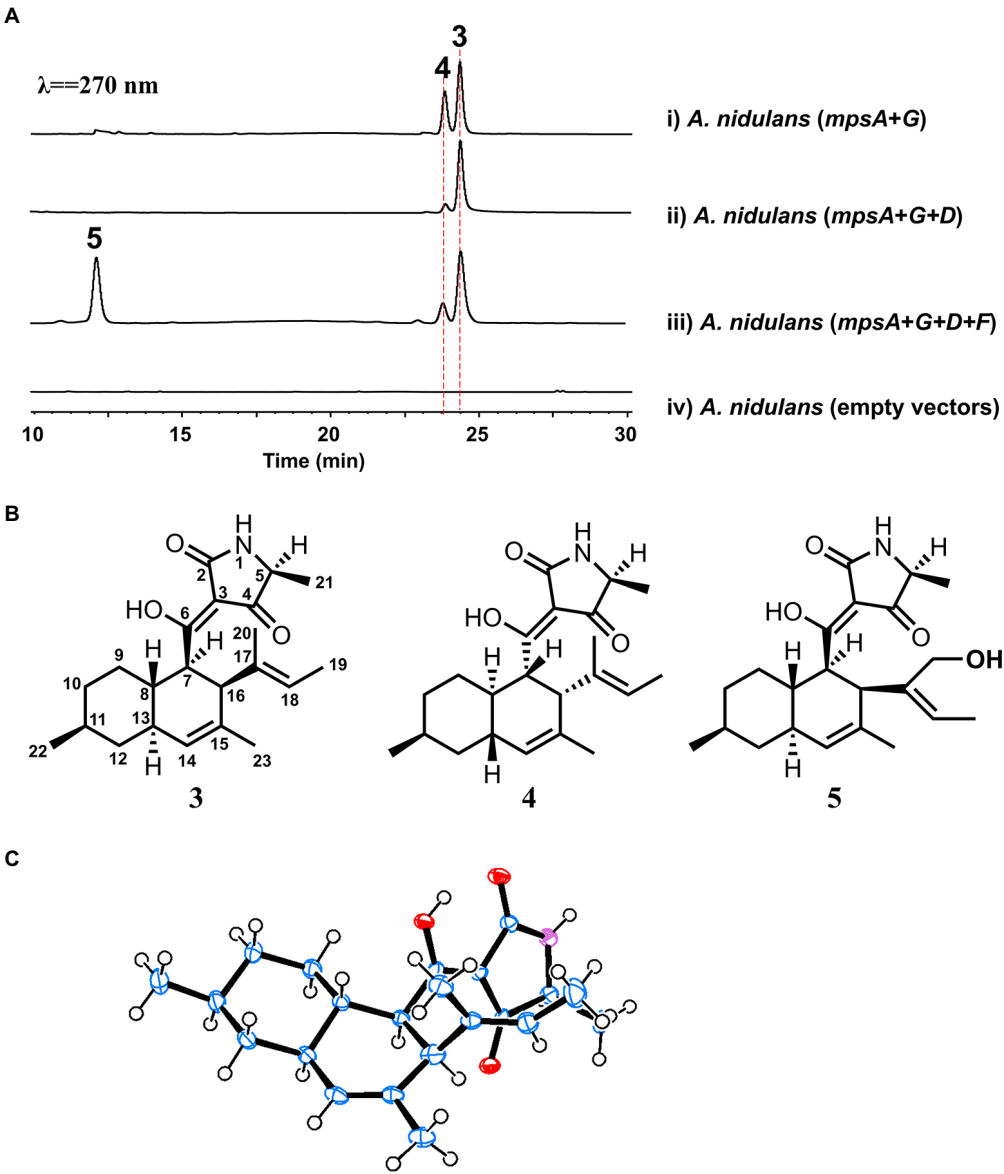
Biosynthesis of macrophasetins. **(A)** Metabolites analysis of heterologous reconstitution of the *mps* cluster in *A. nidulans*. The traces are HPLC with λ = 270 nm. **(B)** The structures of the characterized compounds. **(C)** Crystal structure of **3**.

To explore the natural product encoded by the *mps* cluster, we introduced all the four biosynthetic genes, *mpsA*, *mpsD*, *mpsF* and *mpsG*, into the heterologous expression host *Aspergillus nidulans* (Li et al., 2016) on three vectors. Compared to the extract of the control strain harboring three empty vectors, three new metabolites (the major two are **3** and **5**, the minor one is **4**) were accumulated in the extract of *A. nidulans* expressing *mpsADFG* (Figure 3A, trace iii). Of which, **3** and **4** showed the same molecular weight (MW) of 357, and **5** showed the MW of 373 (Supplementary Figure S2). These compounds were isolated and characterized by 1D and 2D NMR spectroscopy to be DTAs (Supplementary Tables S2–S4; Supplementary Figures S3–S20), which is consistent with the bioinformatics analysis results of *mps* cluster.

For instance, the ^1^H NMR spectrum of **3** showed five methyl groups [*δ*_H_ 0.95 (d, *J* = 7.2 Hz), 1.33 (d, *J* = 6.9 Hz), 1.47 (overlapped), 1.48 (brs), and 1.51 (s)], two olefinic protons [*δ*_H_ 5.23 (s), and 5.10 (d, *J* = 6.5 Hz)], and a series of aliphatic multiplets. The ^13^C NMR of **3** combined with DEPT experiment resolved 22 carbon signals attributable to a ketocarbonyl (*δ*_C_ 195.7), an amide carbonyl (*δ*_C_ 175.4), three *sp*^2^ quaternary carbons (*δ*_C_ 101.2, 133.1 and 134.9), two *sp*^2^ methines (*δ*_C_ 123.3 and 128.4), an oxygenated *sp*^2^ quaternary carbons (*δ*_C_ 191.7), five methyls, three *sp*^3^ methylenes, and six *sp*^3^ methines (one ammoniated; Supplementary Table S2). As five of eight indices of hydrogen deficiency (IHDs) were accounted for by two carbonyls and three double bonds, the remaining three IHDs required that **3** was tricycle. Above information was similar to those of equisetin (Yin et al., 2013), except for the replacement of an oxygenated methylene and nitrogen methyl in equisetin by a methyl and proton in **3**, respectively. Detailed 2D NMR analyses (^1^H−^1^H COSY, HSQC and HMBC) permitted the establishment of the gross structure of **3** as depicted in Figure 3B.

Based on NOESY, the decalin rings in these three compounds (**3**–**5**) are all in *trans* configuration. In addition, we obtained an X-ray crystal structure of **3** (Figure 3C, CCDC 2210452), which allowed us to confirm the absolute stereochemistry of these three compounds. As for compound **3**, the absolute configuration is 5*S*, 7*R*, 8*S*, 11*S*, 13*S*, 16*S*. As shown in Figure 3B, **3** and **5** shared the same scaffold, the difference is that in **5** there is an additional hydroxyl group at C20. While, in compound **4**, the stereochemistry of the decalin ring is different from **3** and **5**. After searching in the compound databases, all the three compounds are novel DTAs, named macrophasetin A (**3**), B (**4**), and C (**5**). Both **3** and **5** exhibited antimicrobial activities against *Bacillus subtilis*, inhibiting the growth of *B. subtilis* at the concentration of 10 μg/ml (Supplementary Table S5). These results confirmed our hypothesis that the *mps* cluster could encode novel DTAs. Combined the results from the structural elucidation and the cluster bioinformatics analysis, we proposed that **5** is the end product of *mps* cluster.

### Biosynthesis of macrophasetins

**Figure 4:**
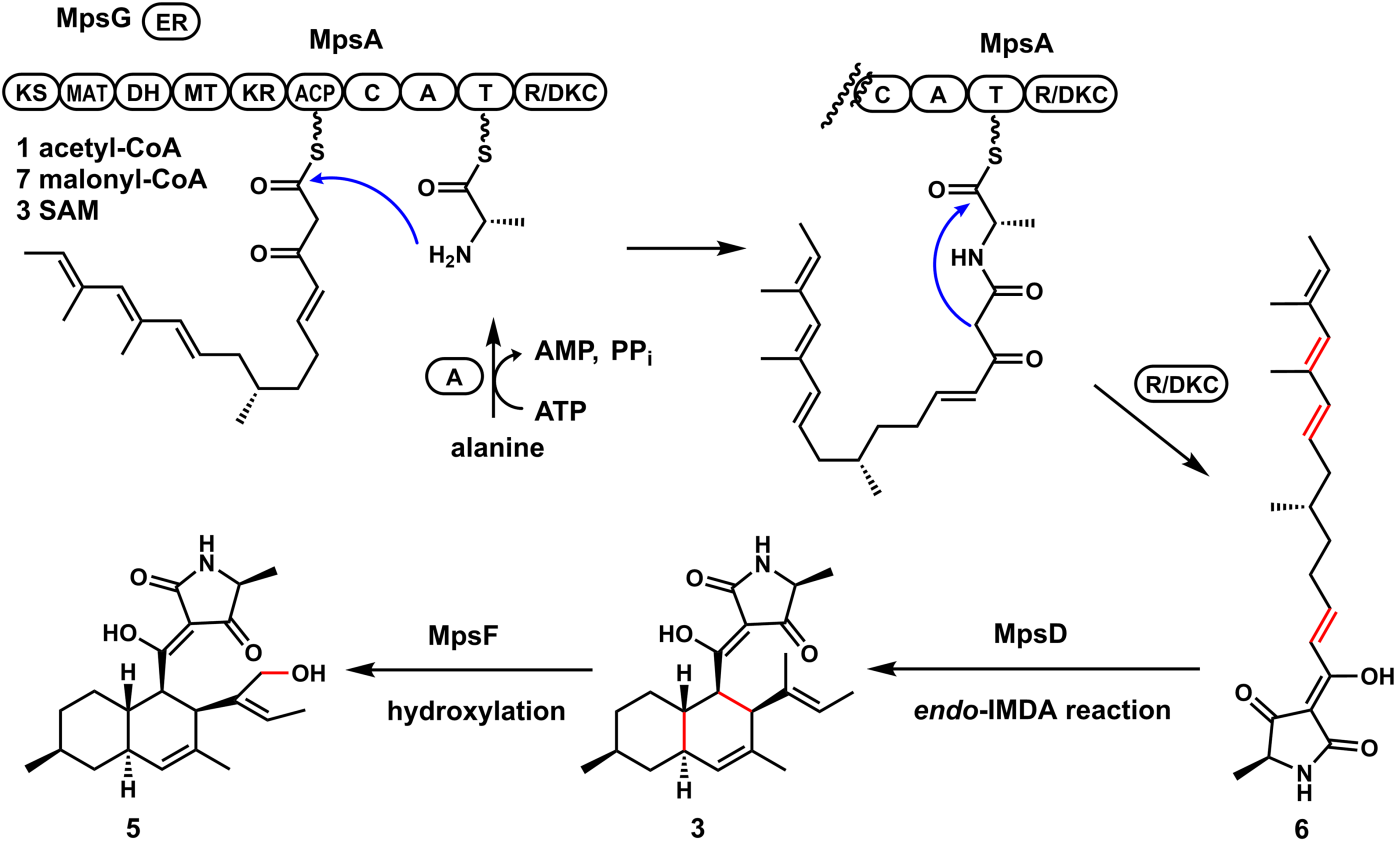
The proposed biosynthetic pathway of macrophasetins.

To further validate the proposal and investigate the biosynthesis of macrophasetins, the heterologous expression of combinations of *mps* cluster genes was carried out. When the PKS-NRPS MpsA and its associated enoylreductase partner MpsG were co-expressed in *A. nidulans*, the expected linear tetramic acid product **6** (structure shown in Figure 4) was not detected from the extracts of the co-expression strain, instead of the decalin-containing compounds **3** and **4** (Figure 3A, trace i). We proposed that the formation of **3** and **4** resulted from the spontaneous non-enzymatic IMDA reaction of **6**. However, the results still validated the predicted function of MpsA and MpsG, since the structure of **6** is almost identical to the predicted tetramic acid **1**. The only difference is the position of one methyl group, in compound **1** the methyl group attached to carbon 9 while in **6** it attached to carbon 15. This result further proofed our hypothesis that the closer the phylogenetic relationship of PKS domains the closer the structure of their synthesized polyketides.

When MpsA/G and the putative LLDAse MpsD were co-expressed in *A. nidulans*, **3** was detected from the extract of co-expression strain as the major product (Figure 3A, trace ii). Though a little amount of **4** was still detected, the ratio of **3**/**4** changed a lot compared to the co-expression strain of MpsA/G. Therefore, the proposed function of MpsD was confirmed to be a DAse catalyzing the *endo*-IMDA reaction of the linear substrate **6** just as predicted. In addition, since the production of **5** was not detected from the strain expressing MpsA/D/G, we concluded that **5** is the end product of the *mps* cluster. And the function of the P450 MpsF was validated to be a hydroxylase catalyzing the hydroxylation on **3** at C20 position.

Based on these findings, we proposed the biosynthetic pathway of macrophasetins. As shown in Figure 4, the PKS-NRPS MpsA together with its associated enoylreductase partner MpsG incorporate one unit of acetyl-CoA, seven units of malonyl-CoA, and one unit of l-alanine to assemble the backbone of macrophasetins to afford the linear tetramic acid intermediate **6**. Without the LLDAse MpsD, **6** can undergo the non-enzymatic IMDA reaction to generate both **3** and **4**. Catalyzed by MpsD, **6** is thoroughly converted to **3** *via* the *endo*-IMDA reaction in a regioselective and stereoselective manner. Finally, the P450 MpsF catalyzes the hydroxylation at C20 to yield the end product **5**.

## Conclusion

In summary, guided by the bioinformatics analysis prediction, we mined a biosynthetic gene cluster from the plant pathogenic fungus *M. phaseolina*, which was characterized to be responsible for the biosynthesis of novel DTAs. We also investigated the biosynthesis of these DTAs, and validated the accuracy of the bioinformatics prediction. Our results provide a successful example to the proof of concept that combining phylogeny-guided bioinformatics prediction and heterologous expression is a powerful approach to mine novel DTAs from fungal species, which may facilitate the novel natural products discovery.

## Data availability statement

The datasets presented in this study can be found in online repository. The name of the repository is the Cambridge Crystallographic Data Centre and the accession number is 2210452. The X-ray data of 3 has been deposited to CCDC and released. You can find the information via this link https://www.ccdc.cam.ac.uk/structures/Search?Ccdcid=2210452&DatabaseToSearch=Published.

## Author contributions

LL, MT, and HZ conceived and designed the experiments. CY, LC, YG, JL, PL, MZ, and QL performed the experiments. LC, CY, MT, and LL analyzed the data. CY, MT, and LL interpreted the results. MT and LL wrote the manuscript. All authors contributed to the article and approved the submitted version.

## Funding

This work was supported by the National Key R&D Program of China under Grant nos. 2021YFA0910501 and 2018YFA0901900, the National Natural Science Foundation of China under Grant nos. 31870039 and 32170069, and the Natural Science Foundation of Fujian Province under Grant no. 2021J01173.

## Conflict of interest

The authors declare that the research was conducted in the absence of any commercial or financial relationships that could be construed as a potential conflict of interest.

## Publisher’s note

All claims expressed in this article are solely those of the authors and do not necessarily represent those of their affiliated organizations, or those of the publisher, the editors and the reviewers. Any product that may be evaluated in this article, or claim that may be made by its manufacturer, is not guaranteed or endorsed by the publisher.

## Supplementary material

The Supplementary material for this article can be found online at: https://www.frontiersin.org/articles/10.3389/fmicb.2022.1056392/full#supplementary-material
