# Supplementary material for: Discovery and biosynthesis of macrophasetins from the plant pathogen fungus Macrophomina phaseolina
Source: Front Microbiol. 2022 Nov 14;13:1056392. doi: 10.3389/fmicb.2022.1056392 (PMC9701702; doi:10.3389/fmicb.2022.1056392)
Supplement: Supplementary file 1 [file Data_Sheet_1.pdf]

SUPPORTING INFORMATION

Discovery and Biosynthesis of Macrophasetins from the Plant Pathogen Fungus *Macrophomina phaseolina*

Cui Yu,[1, ‡] Lin Chen,[2, 3, ‡] Yang-Le Gao,[1] Jia Liu,[1] Pei-Lin Li,[1] Ming-Liang Zhang,[1] Qin Li,[1] Huai-Dong Zhang,[1, *] Man-Cheng Tang,[2,3, *] Li Li[1, *]

1 Engineering Research Center of Industrial Microbiology (Ministry of Education) and College of Life Sciences, Fujian Normal University, Fuzhou 350117, China;

2 State Key Laboratory of Microbial Metabolism, Joint International Research Laboratory of Metabol-ic & Developmental Sciences, School of Life Sciences and Biotechnology, Shanghai Jiao Tong University, Shanghai 200240, China;

3 Zhangjiang Institute for Advanced Study, Shanghai Jiao Tong University, Shanghai 200240, China.

Table **S1** Primers used in this study

Primer	Sequence (5'→3')
UmpsAF1	CTTCATCCCCAGCATCATTACACCTCAGCATTAATTAAATGCCT CAACGCAACGAGCCC
UmpsAR1	TCGAGCGTCTTGAAGATCATG
UmpsAF2	TGGCACTGGTGGTTCTACCAG
UmpsAR2	AGTGCGGCCAGGTAGAAGTGG
UmpsAF3	GTACGCCGAAGTGTCGGCGAC
UmpsAR3	TTGTTATATCATTTATAGCTCGTTCGGCACCTTTAATCGTGGAA ATGGTGCGTGTCAAT
AmyB-pYTU-F	CAGCGAACACGCCAGAAATTGACACGCACCATTTCCACGATTA AAGGTGCCGAACGAGC
AmyB-pYTU-R	AATGTGAGGGCCGTCTGTTTTGCGGGGAGGTCTGCCATAAATG CCTTCTGTGGGGTTTA
UmpsGF	TCCCTTCTCTGAACAATAAACCCCACAGAAGGCATTTATGGCAG ACCTCCCCGCAAAAC
UmpsGR	AGTGGAGGACATACCCGTAATTTTCTGGGCATTTAAATAGCCGC GGTACACGGGCCATG
PmpsCF	CTCTGAACAATAAACCCCACAGAAGGCATTTTTAATTAAATGG CACATCCGCAGAGCC
PmpsCR	GATGAGACCCAACAACCATGATACCAGGGGATTTAAATCGCGG ACCGACCATGTCTTGG
RmpsDF	GACTAACCATTACCCCGCCACATAGACACATCTAAACAATGGA CGTCTCGAATGGTTCA
RmpsDR	TAAAGGGTATCATCGAAAGGGAGTCATCCAATTTAAATGCATG CAGTGATGAACTAATG

Table **S2.** ^{1}H (600 MHz) and ^{13}C (150 MHz) NMR data of **3** in $CDCl_3$ (δ in ppm).

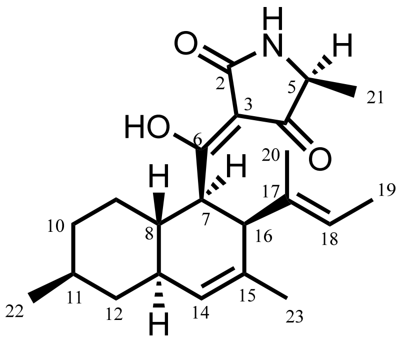

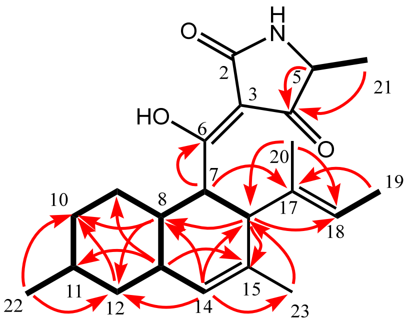

Key ^{1}H-^{1}H COSY (bold lines) and HMBC (red →) correlations of **3**

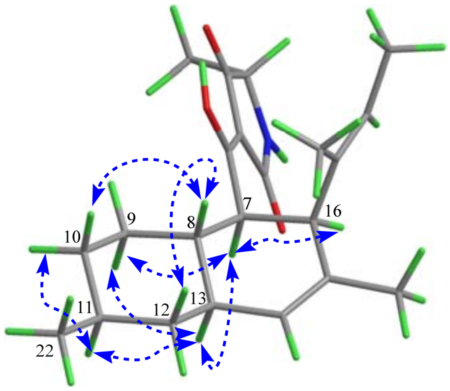

Key NOE (blue dashed arrows) correlations of **3**

"m" means overlapped or multipet with other signals.

No.	δ_H , mult (*J* in Hz)	δ_C, type
2		175.4, C
3		101.2, C
4		195.7, C
5	3.84, q (6.9)	57.7, CH
6		191.7, C
7	3.68, dd (12.1, 6.5)	47.9, CH
8	1.62, m	37.4, CH
9	1.53, m	25.0, CH_2
	1.04, m	
10	1.56, m	31.9, CH_2
	1.45, m	
11	2.03, m	27.6, CH
12	1.51, m	38.9, CH_2
	1.34, m	
13	1.96, m	35.2, CH
14	5.23, s	128.4, CH
15		133.1, C
16	2.88, d (6.4)	52.1, CH
17		134.9, C
18	5.10, q (6.5)	123.3, CH
19	1.48, brs	13.5, CH_3
20	1.51, s	15.0, CH_3
21	1.33, d (6.9)	17.4, CH_3
22	0.95, d (7.2)	18.4, CH_3
23	1.47, m	21.9, CH_3

Table **S3.** ^{1}H (600 MHz) and ^{13}C (150 MHz) NMR data of **4** in CD_3OD (δ in ppm).

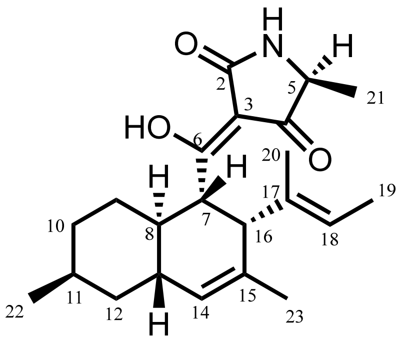

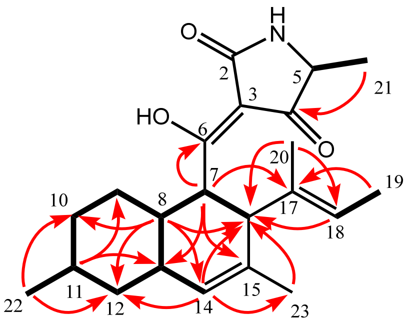

Key ^{1}H-^{1}H COSY (bold lines) and HMBC (red →) correlations of **4**

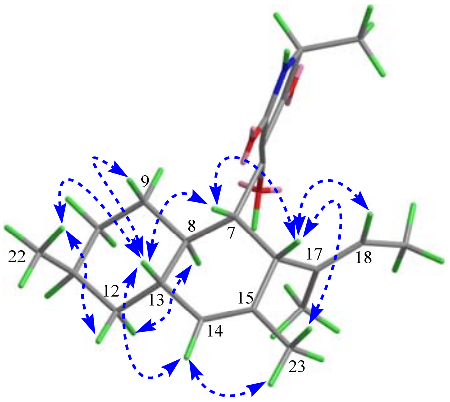

Key NOE (blue dashed arrows) correlations of **4**

“m” means overlapped or multipet with other signals.

No.	δ_H , mult (J in Hz)	δ_C, type
2		176.6, C
3		102.2, C
4		198.5, C
5	3.90, brs	59.0, CH
6		193.3, C
7	3.71, dd (10.6, 6.5)	49.9, CH
8	1.68, m	38.9, CH
9	1.62, m	26.2, CH_2
	1.07, m	
10	1.58, m	33.1, CH_2
	1.53, m	
11	2.06, m	29.1, CH
12	1.62, m	40.2, CH_2
	1.41, m	
13	1.99, m	36.7, CH
14	5.28, s	129.7, CH
15		134.4, C
16	2.95, brs	53.4, CH
17		136.4, C
18	5.14, q (6.4)	124.5, CH
19	1.52, m	13.7, CH_3
20	1.55, s	15.0, CH_3
21	1.31, d (7.0)	17.6, CH_3
22	1.03, d (7.2)	18.8, CH_3
23	1.51, m	22.3, CH_3

Table **S4.** ^{1}H (600 MHz) and ^{13}C (150 MHz) NMR data of **5** in DMSO-d_6(δ in ppm).

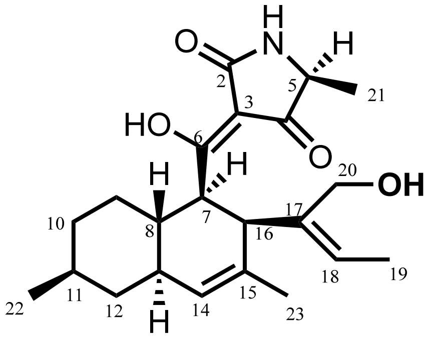

No.	δ_H , mult (J in Hz)	δ_C, type
2		174.9, C
3		101.2, C
4		195.5, C
5	3.84, q (7.0)	57.1, CH
6		190.3, C
7	3.11, m	45.9, CH
8	1.72, m	41.5, CH
9	1.65, m	29.0, CH_2
	0.81, m	
10	1.65, m	34.9, CH_2
	0.91, m	
11	1.46, m	32.6, CH
12	1.72, m	41.7, CH_2
	0.75, q (12.6)	
13	1.46, m	34.6, CH
14	5.30, s	127.6, CH
15		133.6, C
16	3.59, brs	46.8, CH
17		138.6, C
18	5.22, q (6.8)	124.8, CH
19	1.62, d (6.8)	13.4, CH_3
20	4.08, brs	59.0, CH_2
	3.34, brs	
21	1.19, d (7.0)	17.1, CH_3
22	0.87, d (6.5)	22.4, CH_3
23	1.52, brs	21.8, CH_3

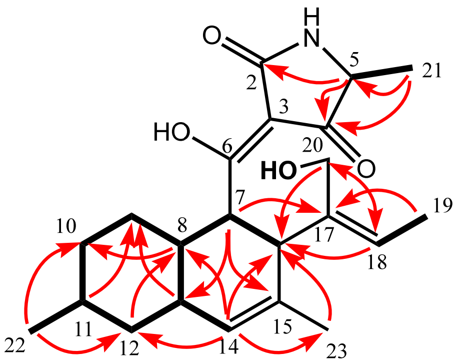

Key ^{1}H-^{1}H COSY (bold lines) and HMBC (red →) correlations of **5**

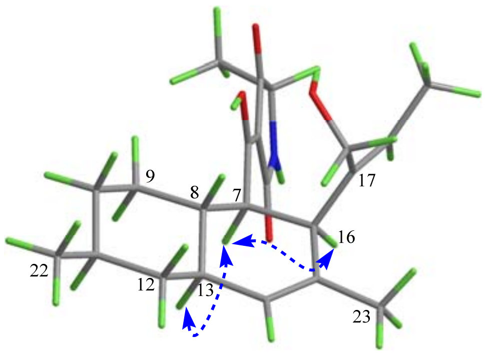

Key NOE (blue dashed arrows) correlations of **5**
“m” means overlapped or multipet with other

Table S5. Antibacterial susceptibility and cytotoxicity profile of Macrophasetin A-C

**Organisms**	**Concentrations (μg·ml^{-1})**			
**Pathogenic bacteria (MIC)**	**Macrosetin A**	**Macrosetin B**	**Macrosetin C**	**Ampicillin**
Staphylococcus aureus ATCC 6538 (MRSA)	>64	>64	>64	2
Staphylococcus aureus ATCC 25923	>64	>64	>64	4
Bacillus subtilis ATCC9372	8	16	16	4
Bacillus cereus ATCC 49064	8	8	16	4
Escherichia coli ATCC 25922	>64	>64	>64	8

Table S6. Sequence alignment of MpsA A domain with other fungal PKS-NRPS A domains.

Signature sequence of the amino acid residues in the adenylation domain of MpsA in comparison with other hybrid PKS-NRPS											
PKS-NRPS	Activated substrate	signature sequence position[a]									
		235	236	239	278	299	301	322	330	331	517
MpsA	Alanine	D	K	E	D	V	A	N	L	D	K
PhyS	Alanine	D	F	D	K	F	G	S	I	A	K
PynA	Alanine	D	L	V	Y	L	A	T	V	Q	K
EqxS	Serine	D	F	D	T	F	G	G	I	L	K
FusA	Homoserine	D	M	T	F	S	A	G	I	I	K
MycA	Leucine	D	L	W	G	M	G	C	V	G	K
PvhA	Leucine	D	P	C	V	A	G	V	M	L	K
CcsA	Phenylalanine	D	M	S	E	V	G	C	F	C	K
TenS	Tyrosine	D	M	V	I	C	G	C	A	A	K
DmbS	Tyrosine	D	M	V	I	C	G	C	A	A	K
CpaA	Tryptophan	D	M	A	L	C	G	S	A	C	K

[a].The residue numbering corresponds to gramicidin S synthetase (PheA:1AMU)[1, 2]

[1]. T. Stachelhaus, H. D. Mootz, M. A. Marahiel, Chem. Biol. 1999, 6, 493 -505.
[2]. Conti, E., Stachelhaus, T., Marahiel, M.A. & Brick, P. (1997). Structural basis for the activation of phenylalanine in the nonribosomal biosynthesis of gramicidin S. EMBO J. 16, 4174-4183.

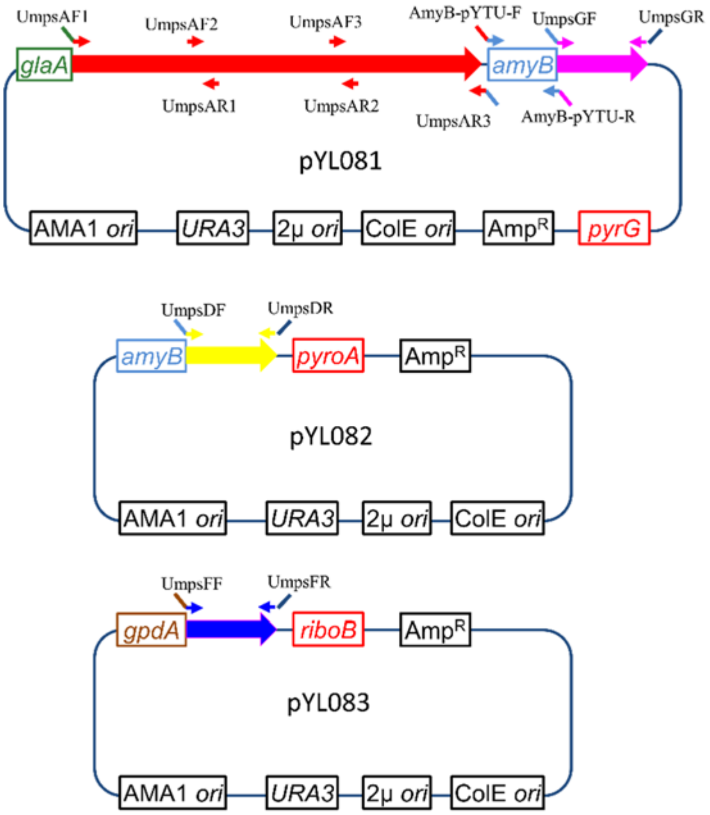

Figure **S1**. Maps of pYL081, pYL082 and pYL083
The primer binding sites were indicated in the maps.

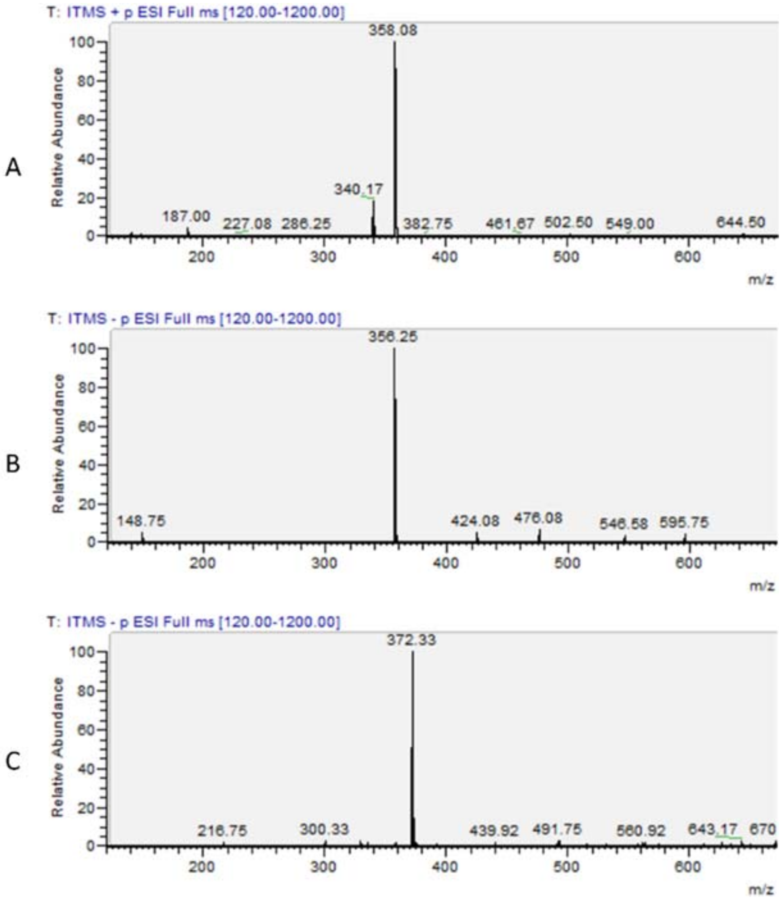

Figure **S2**. MS spectra of **3** (A), **4** (B) and **5** (C).

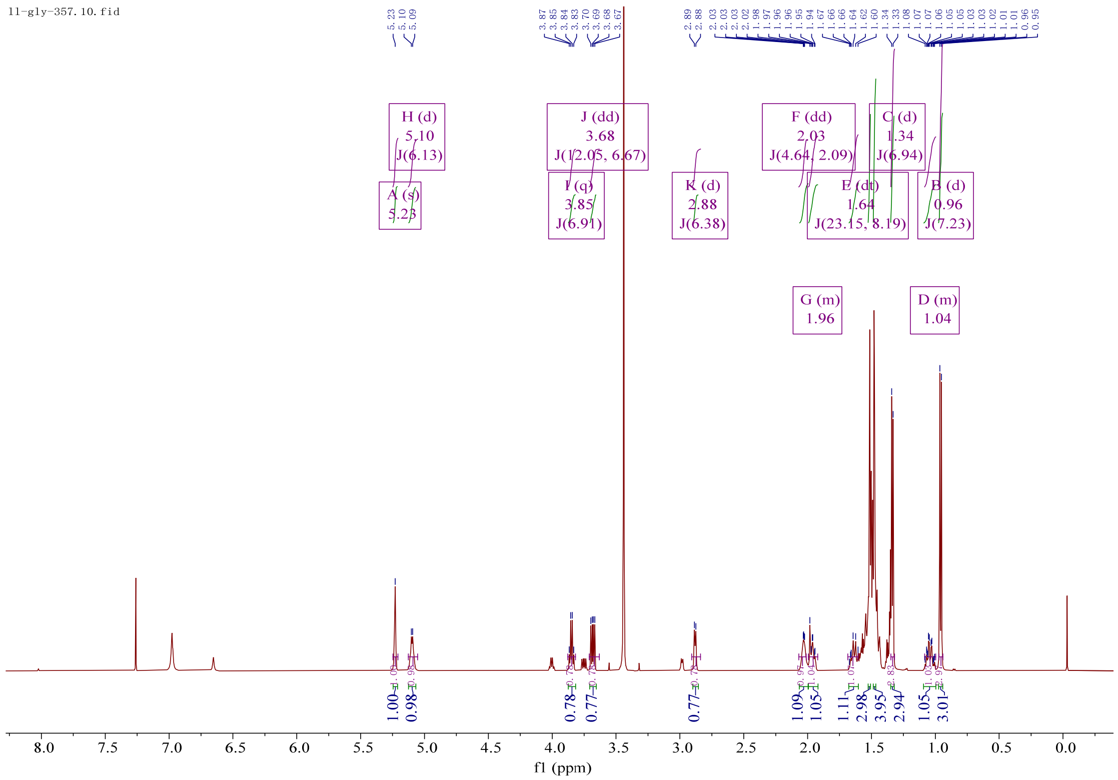

Figure **S3.** ^{1}H NMR of compound **3** in $CDCl_3$.

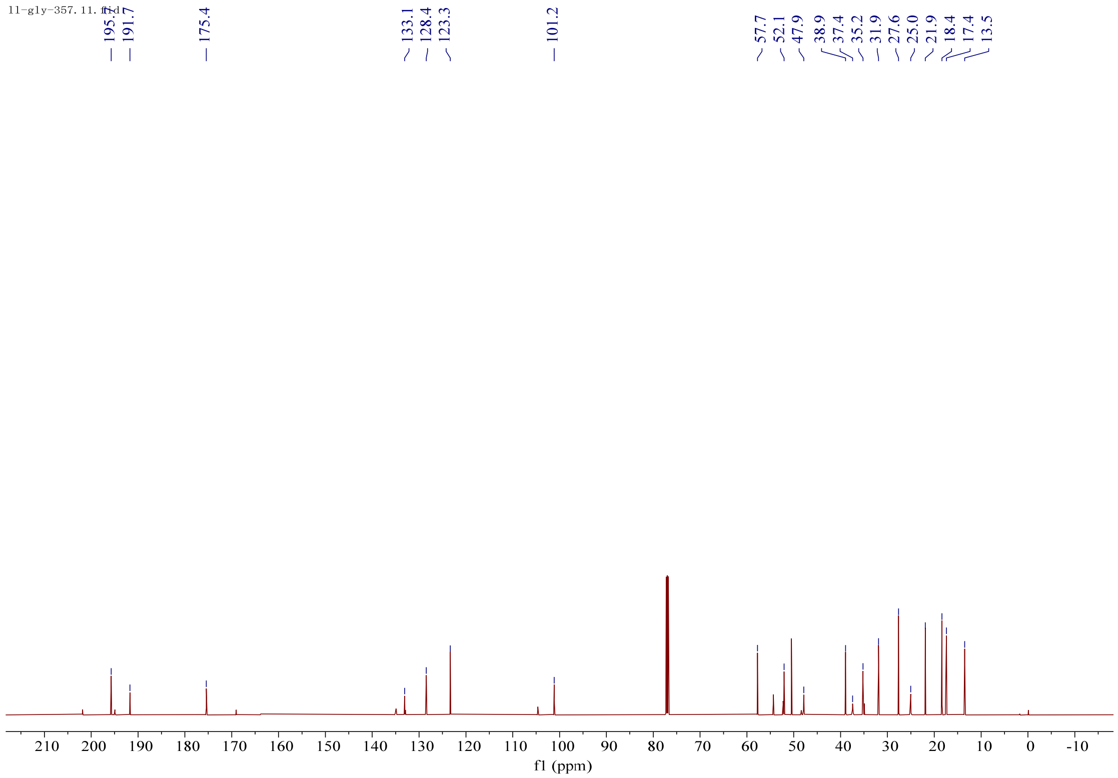

Figure **S4.** ^{13}C NMR of compound **3** in $CDCl_3$.

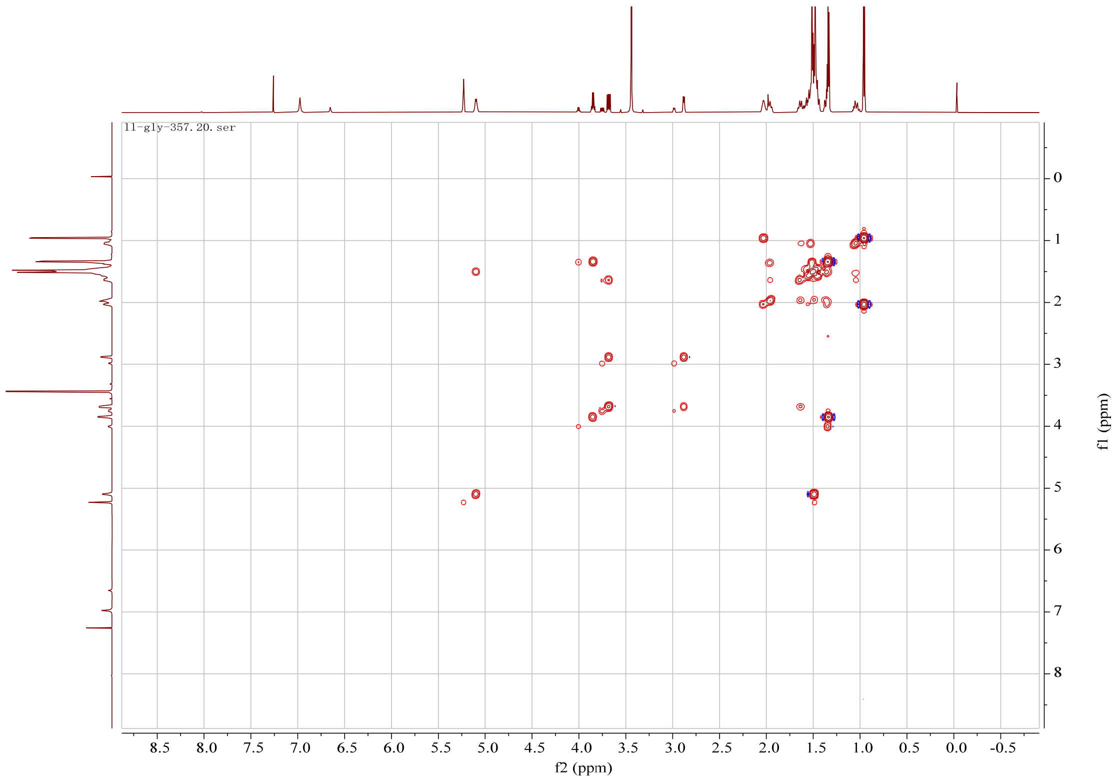

Figure **S5.** COSY of compound **3** in $CDCl_3$.

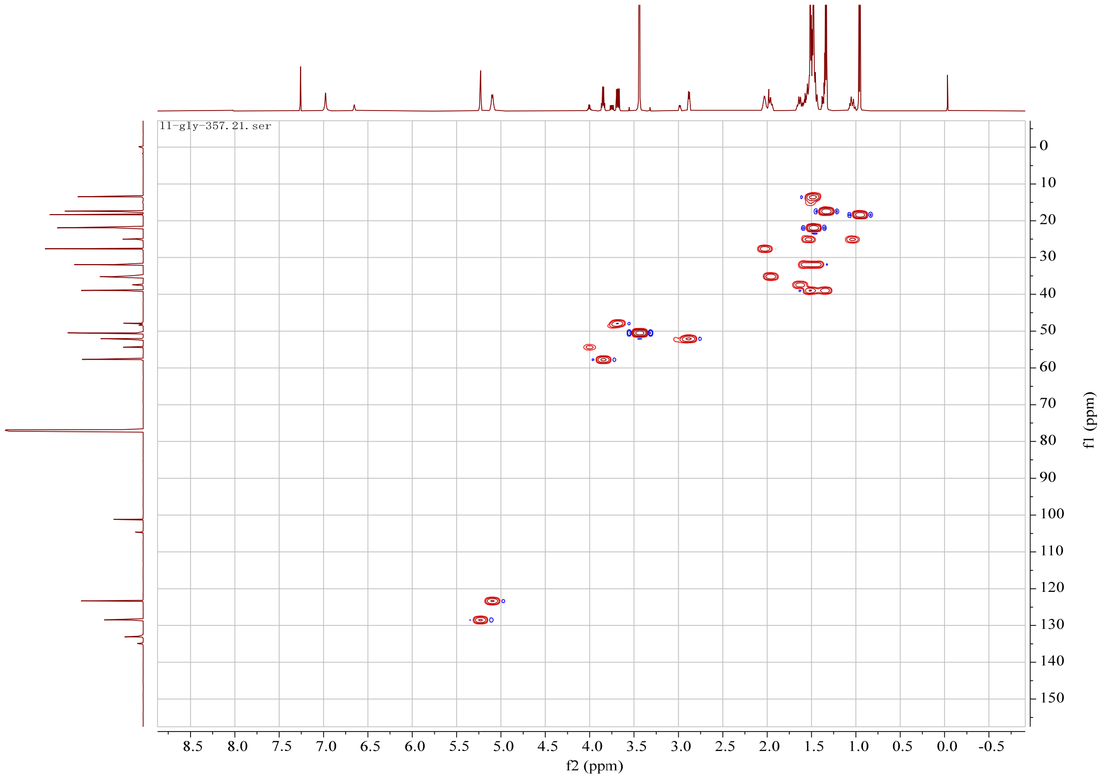

Figure **S6.** HMQC of compound **3** in $CDCl_3$.

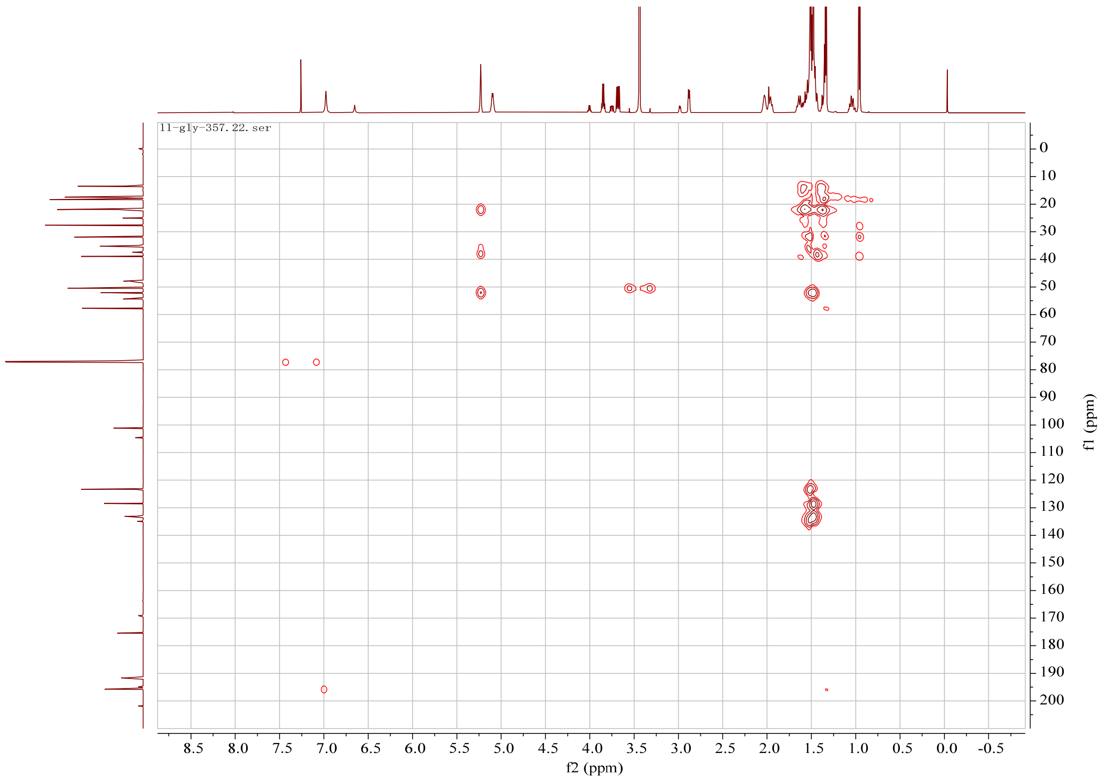

Figure **S7.** HMBC of compound **3** in $CDCl_3$.

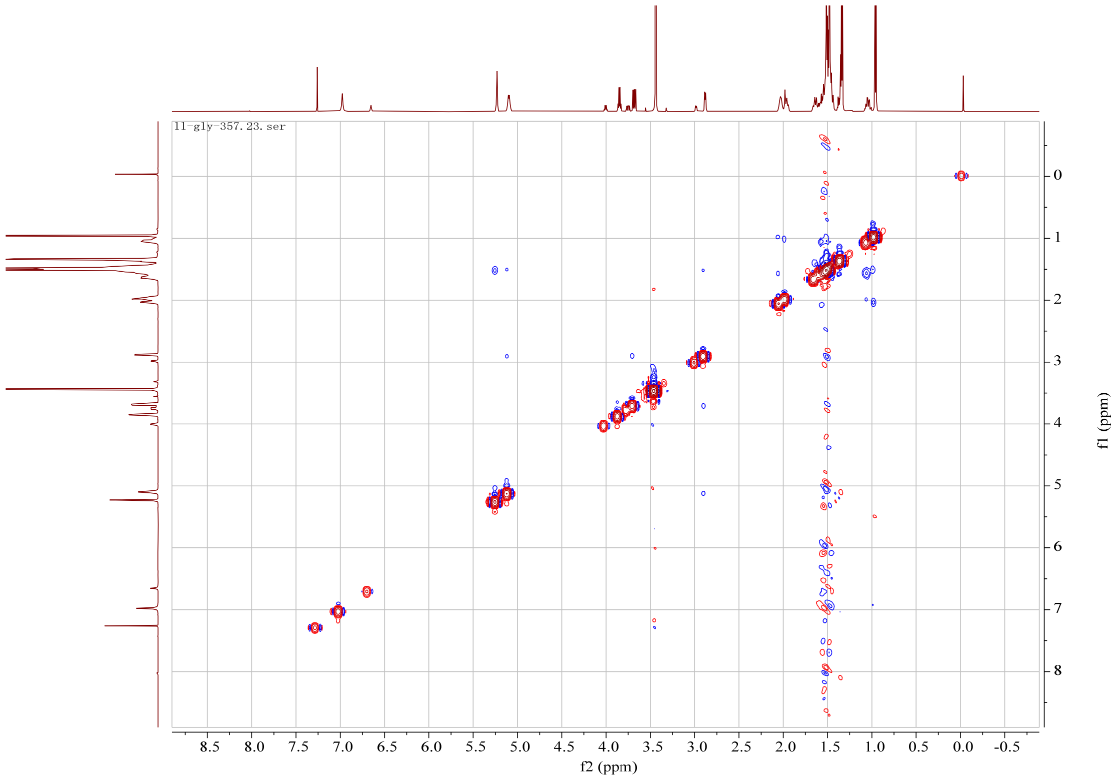

Figure **S8.** NOESY of compound **3** in $CDCl_3$.

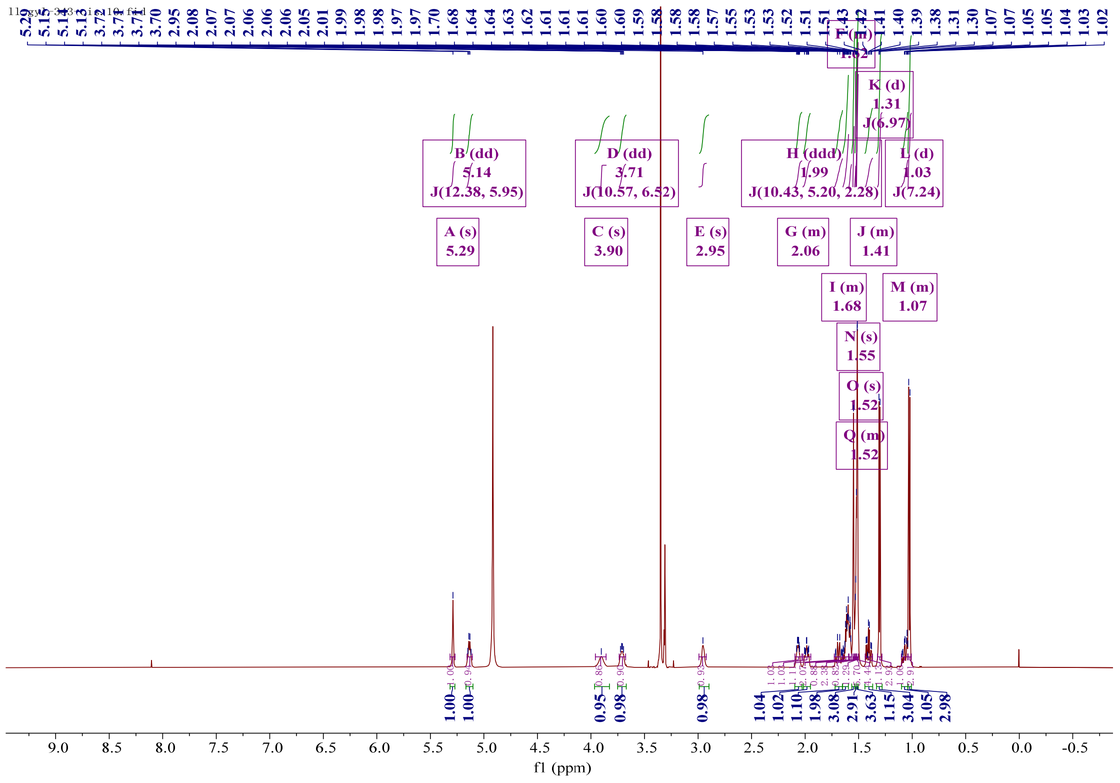

Figure **S9.** ^{1}H of compound **4** in CD_3OD.

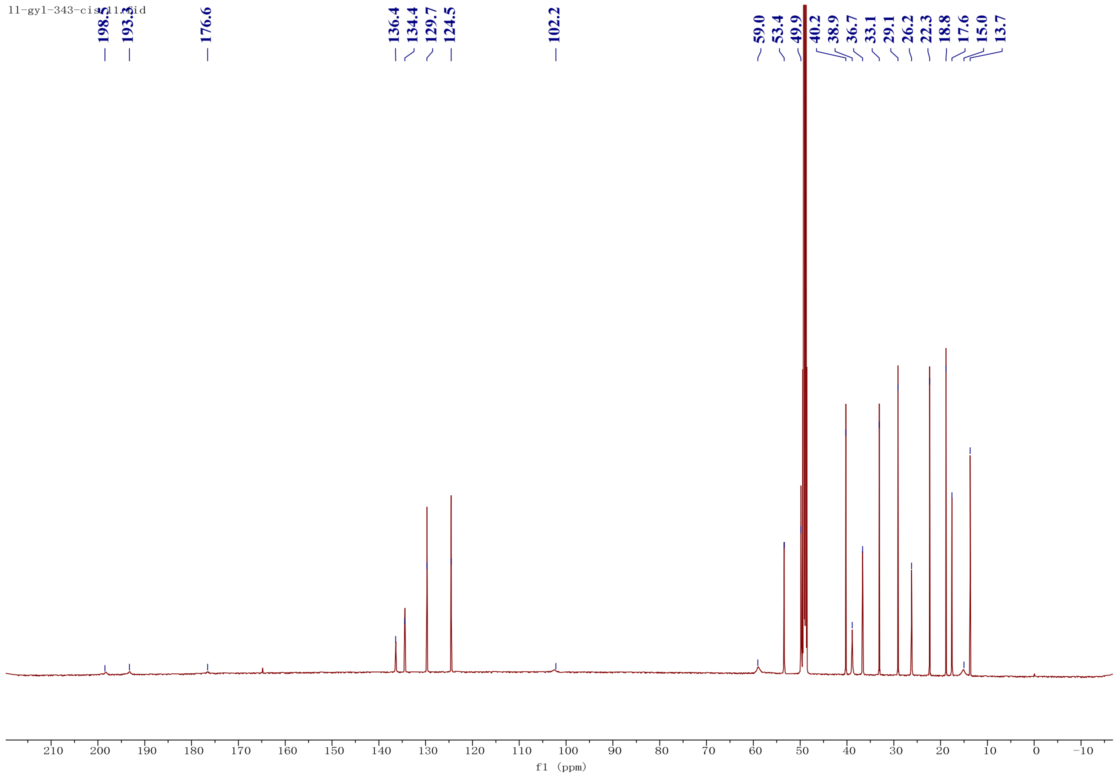

Figure **S10.** ^{13}C of compound **4** in CD_3OD.

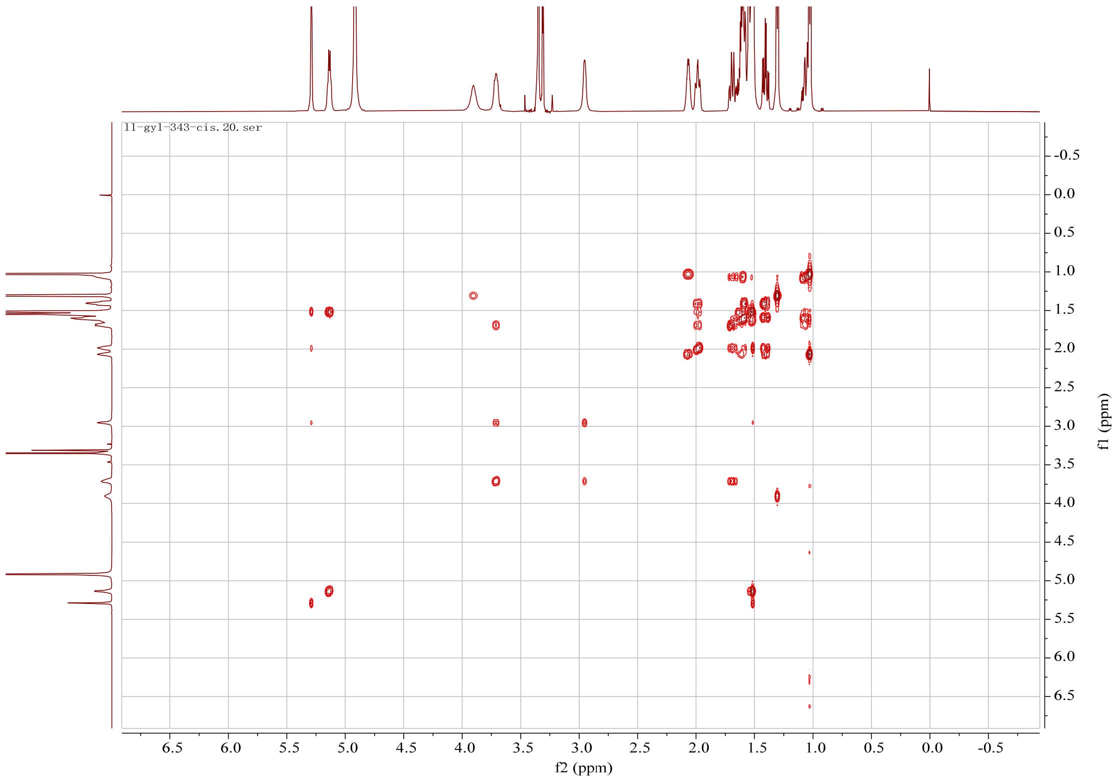

Figure **S11.** COSY of compound **4** in CD_3OD.

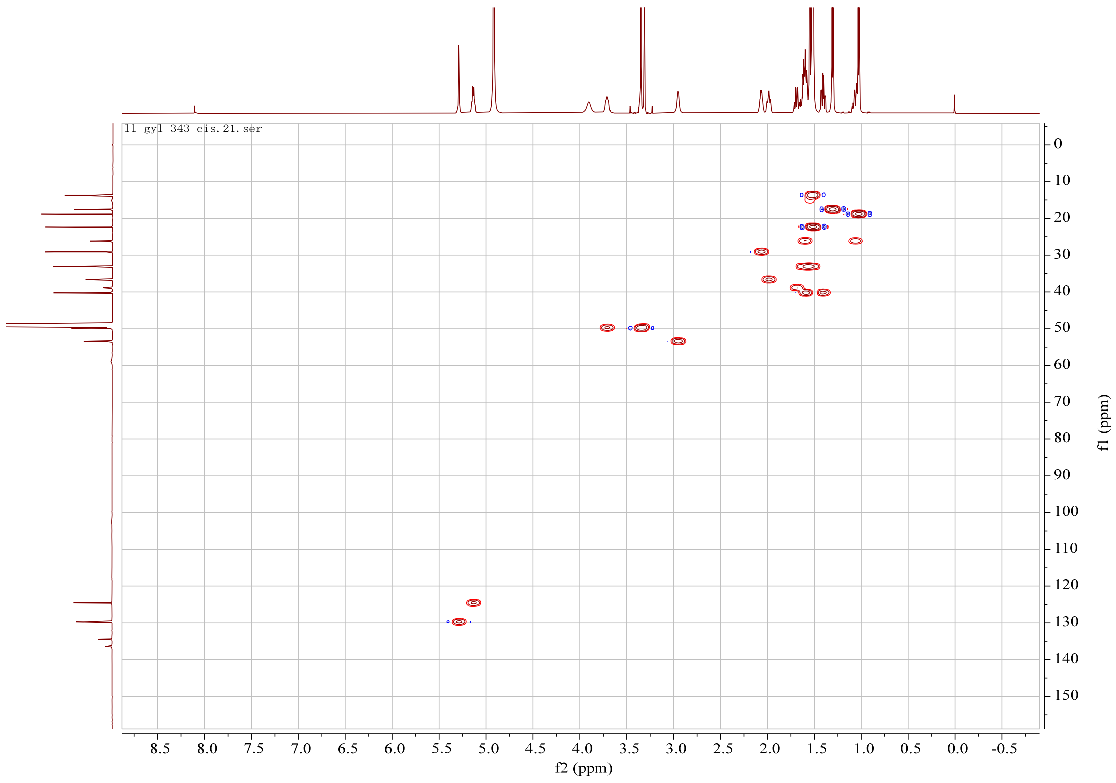

Figure **S12.** HMQC of compound **4** in CD_3OD.

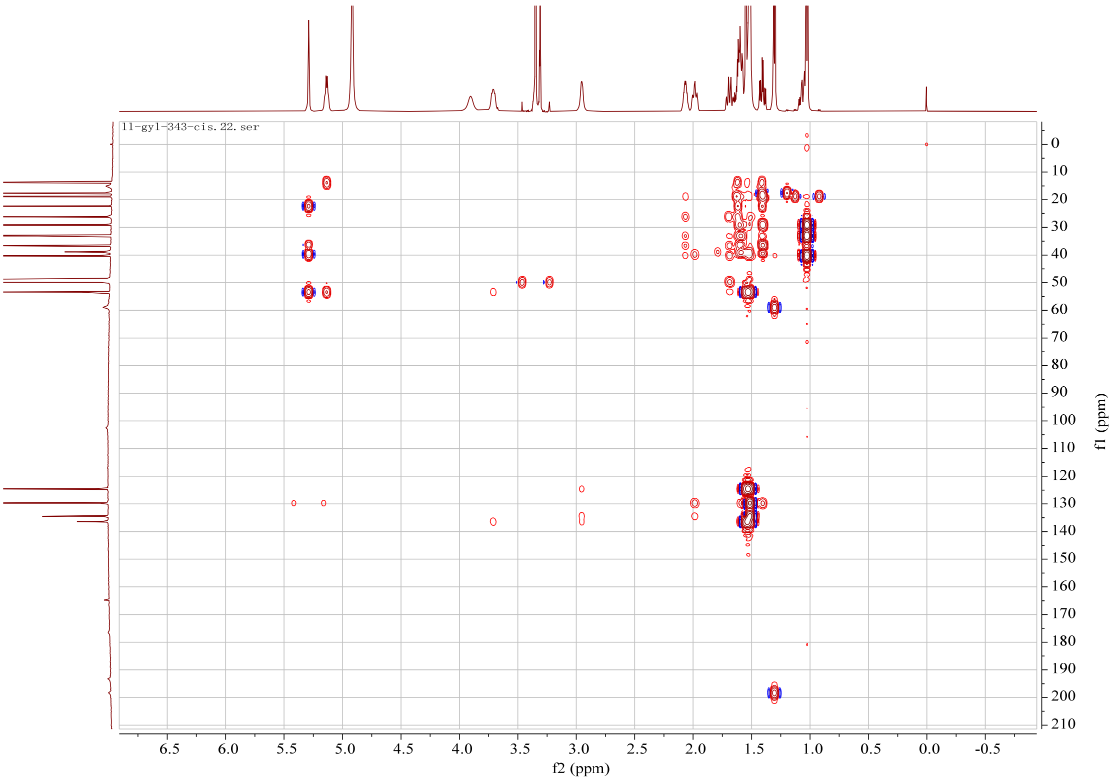

Figure **S13.** HMBC of compound **4** in CD_3OD.

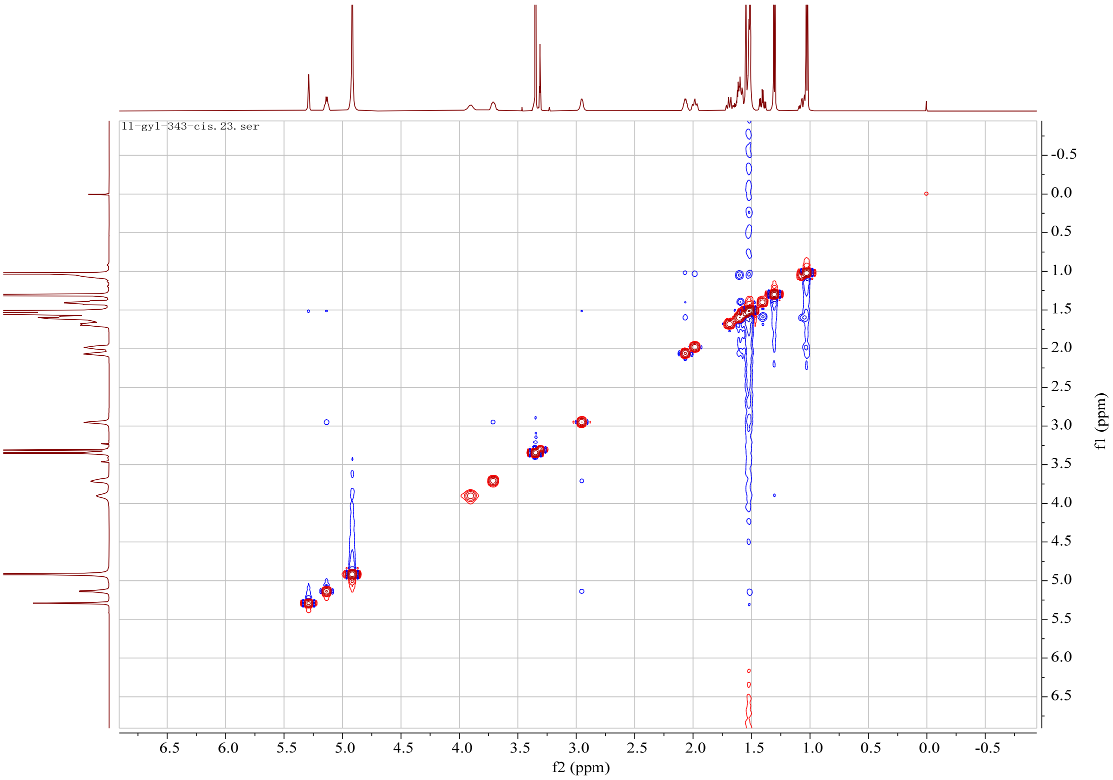

Figure **S14.** NOESY of compound **4** in CD_3OD.

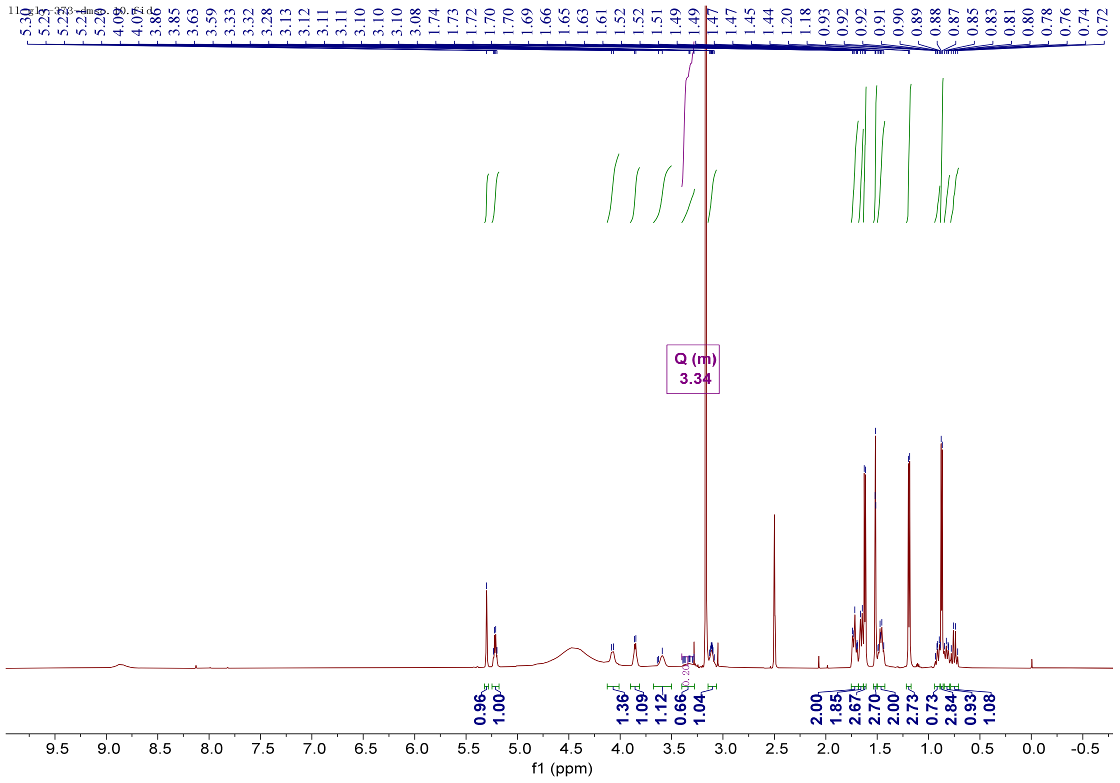

Figure **S15.** ^{1}H of compound **5** in DMSO-d_6.

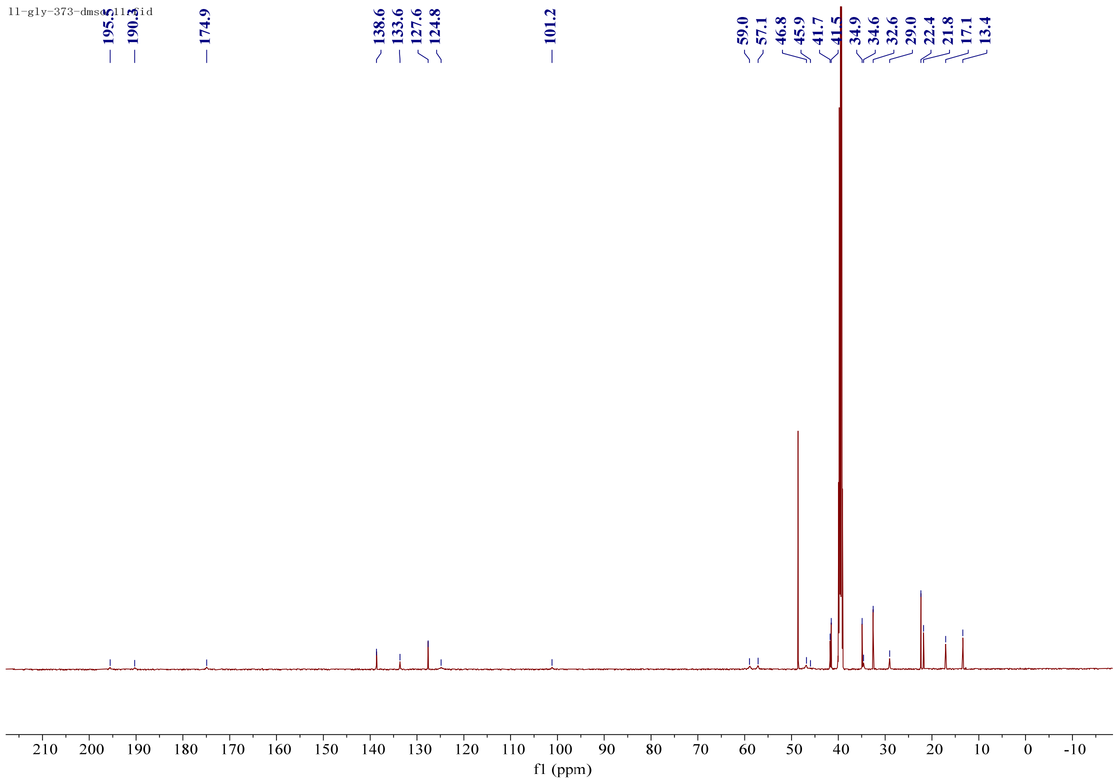

Figure **S16.** ^{13}C of compound **5** in DMSO-d_6.

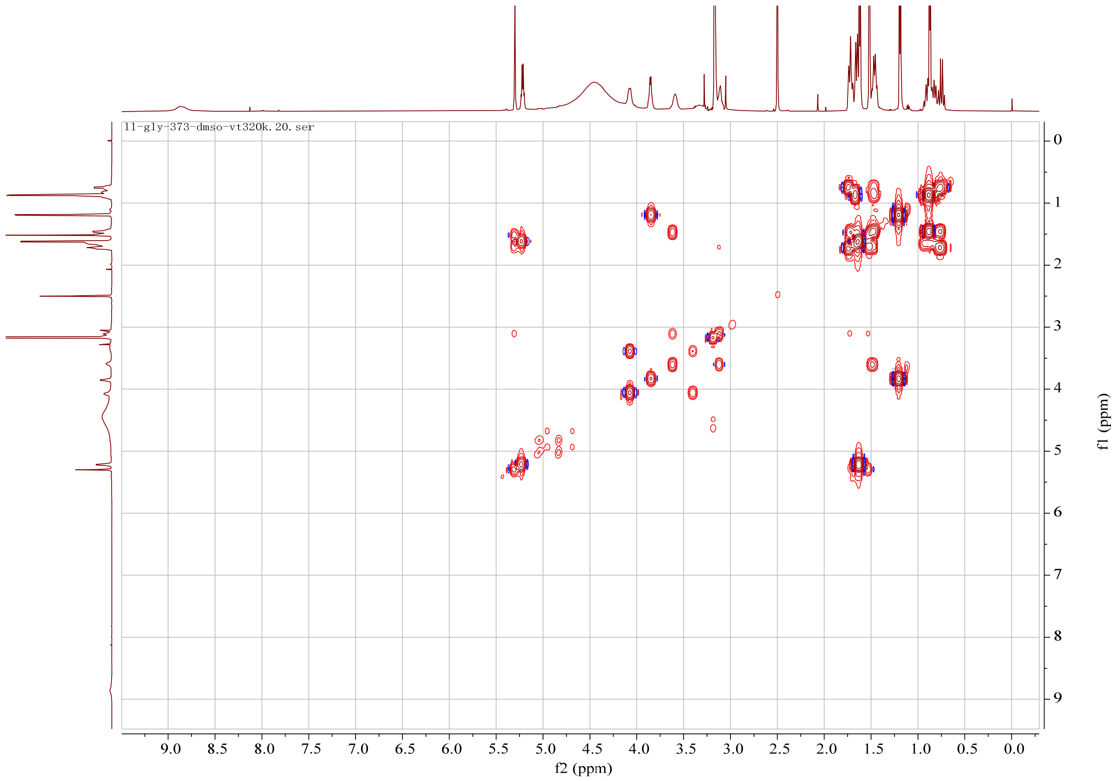

Figure **S17.** COSY of compound **5** in DMSO-d_6.

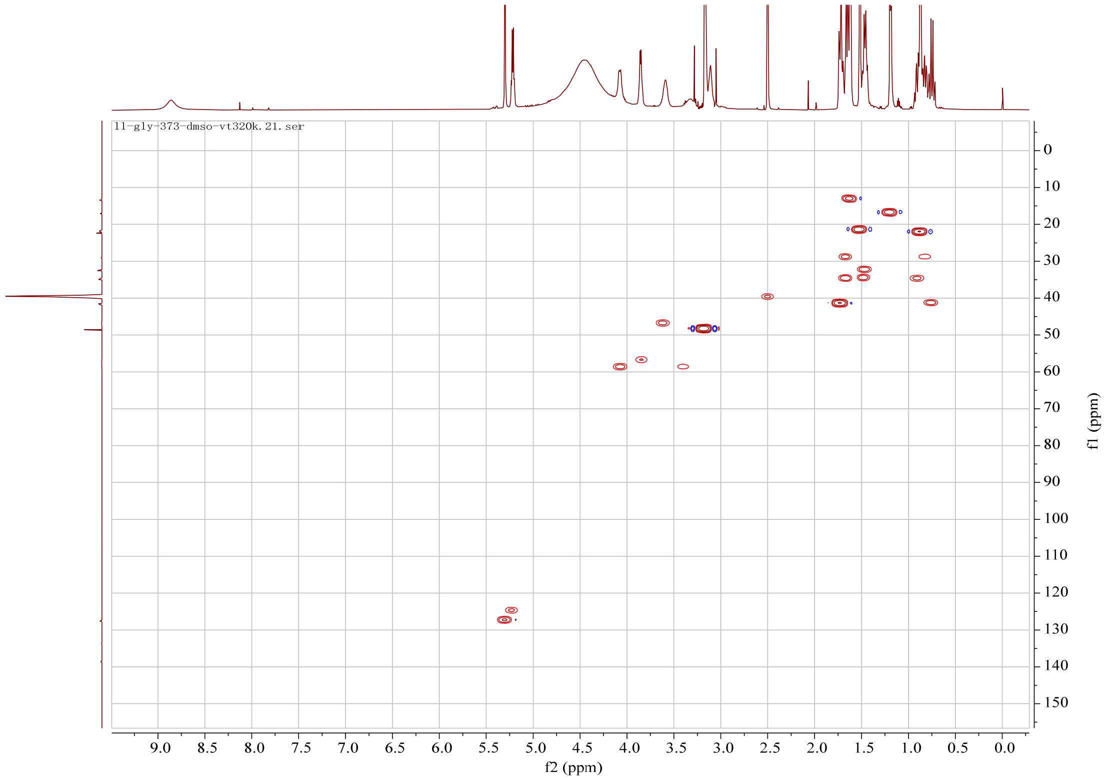

Figure **S18.** HMQC of compound **5** in DMSO-d_6.

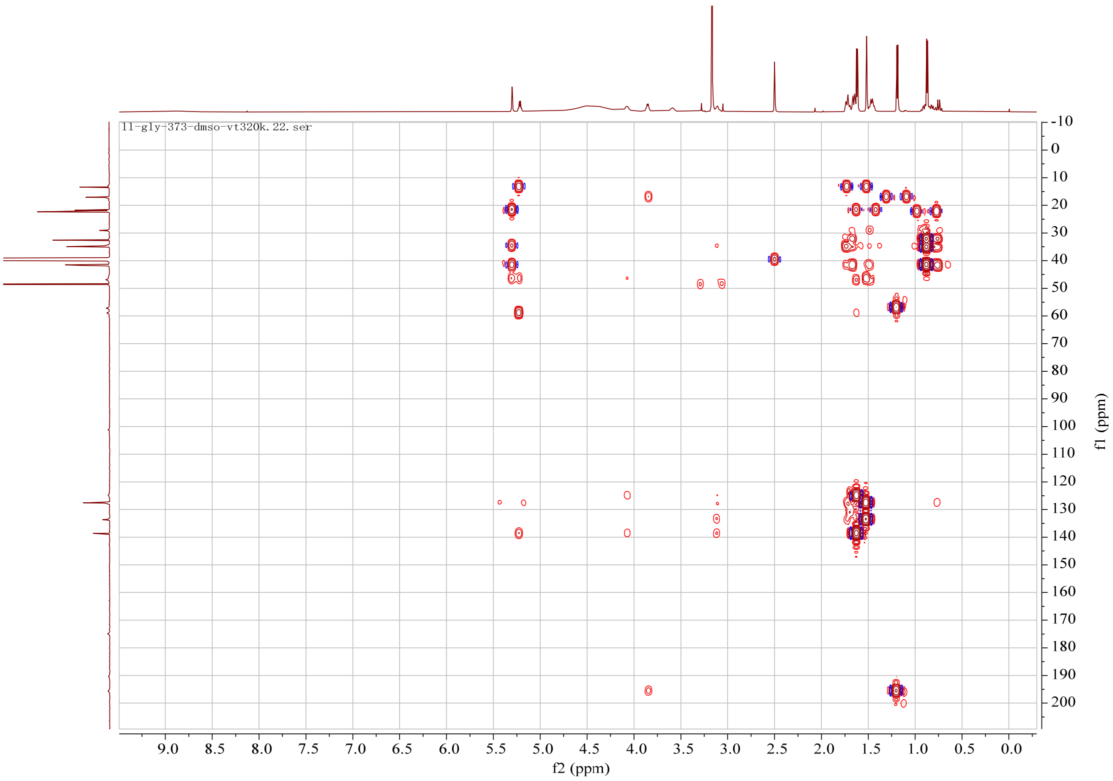

Figure **S19.** HMBC of compound **5** in DMSO-d_6.

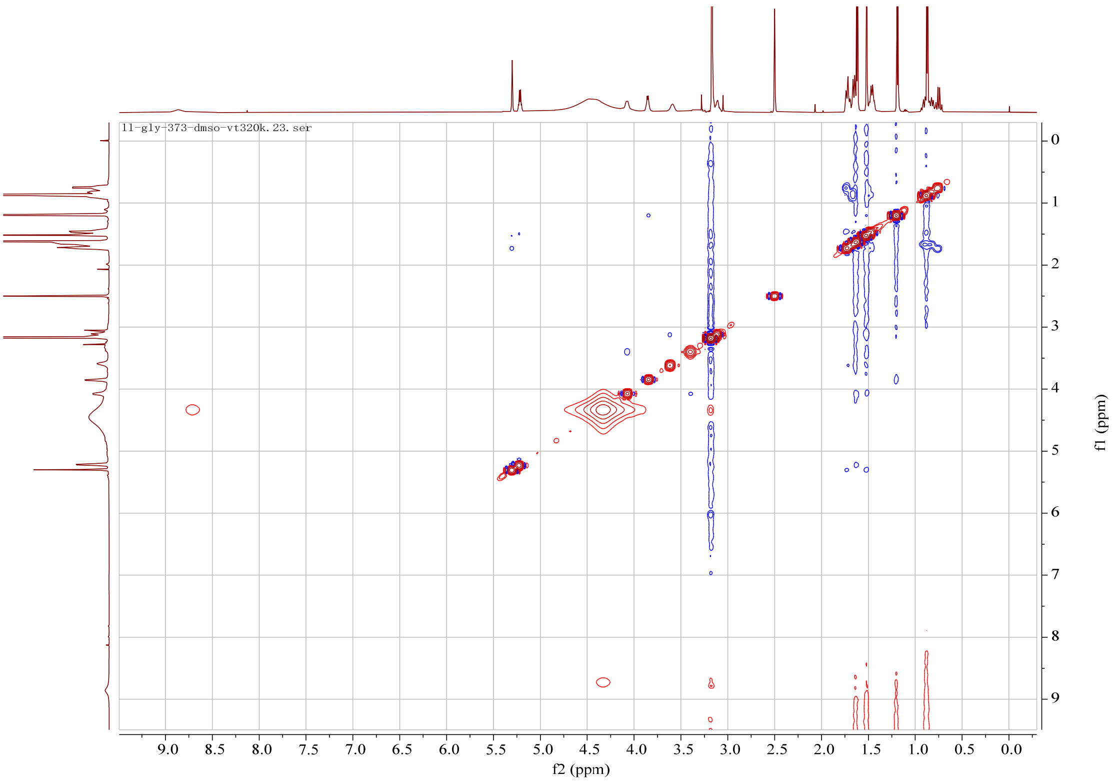

Figure **S20.** NOESY of compound **5** in DMSO-d_6.

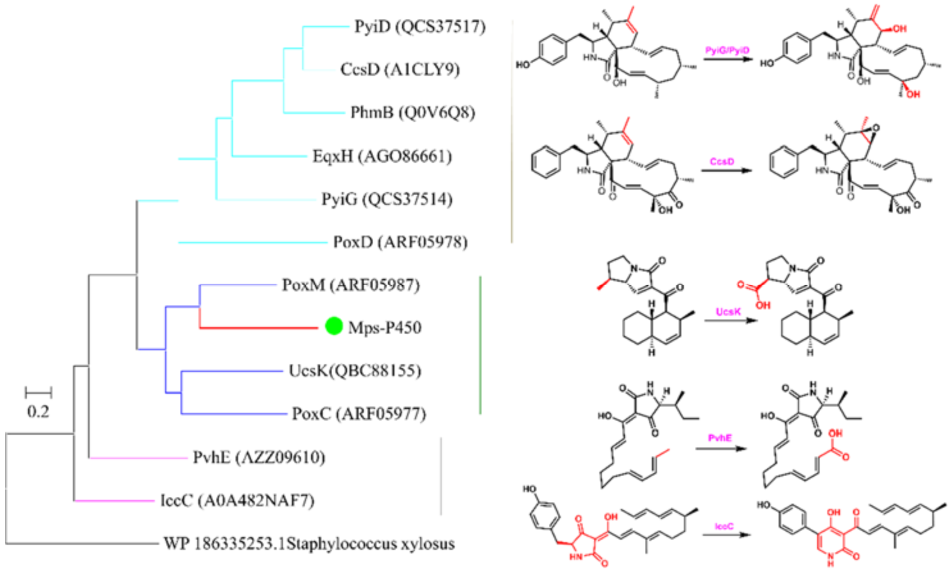

Figure **S21**. Phylogenetic tree analysis of selected P450s involved in the biosynthesis of fungal polyketide-amino acids.

Crystal Data for compound **3**, $C_{22}H_{31}NO_3$ (M = 357.23 g/mol): monoclinic, space group $P2_1$ (no. 4), a = 8.4523(2) Å, b = 11.0390(3) Å, c = 11.7813(4) Å, β = 110.151(3), V = 1031.98(5) Å^3, Z = 2, T = 104(6) K, μ (Cu Kα) = 0.614 mm^{-1}, D_{calc} = 1.157 g/cm^3, 3668 reflections measured (7.994° ≤ 2θ ≤ 151.728°), 2521 unique (R_{int} = 0.0128, R_{sigma} = 0.0198) which were used in all calculations. The final R_1 was 0.0342 (I > 2σ(I)) and wR_2 was 0.0928 (all data). Flack parameter = 0.34 (12). Crystallographic data for the structure of **3** have been deposited in the Cambridge Crystallographic Data Centre (deposition number :CCDC 2210452).
